# Integrative Genomic Analyses Identify *BRF2* as a Novel Lineage-Specific Oncogene in Lung Squamous Cell Carcinoma

**DOI:** 10.1371/journal.pmed.1000315

**Published:** 2010-07-27

**Authors:** William W. Lockwood, Raj Chari, Bradley P. Coe, Kelsie L. Thu, Cathie Garnis, Chad A. Malloff, Jennifer Campbell, Ariane C. Williams, Dorothy Hwang, Chang-Qi Zhu, Timon P. H. Buys, John Yee, John C. English, Calum MacAulay, Ming-Sound Tsao, Adi F. Gazdar, John D. Minna, Stephen Lam, Wan L. Lam

**Affiliations:** 1 Department of Cancer Genetics and Developmental Biology, British Columbia Cancer Research Centre, Vancouver, British Columbia, Canada; 2 Department of Cancer Imaging, British Columbia Cancer Research Centre, Vancouver, British Columbia, Canada; 3 Department of Pathology, Division of Applied Molecular Oncology, University Health Network - Princess Margaret Hospital and Ontario Cancer Institute, University of Toronto, Toronto, Ontario, Canada; 4 Department of Surgery, Vancouver General Hospital, Vancouver, British Columbia; 5 Department of Pathology, Vancouver General Hospital, Vancouver, British Columbia; 6 Hamon Center for Therapeutic Oncology Research, University of Texas Southwestern Medical Center, Dallas, Texas; Vanderbilt University, United States of America

## Abstract

**Background:**

Traditionally, non-small cell lung cancer is treated as a single disease entity in terms of systemic therapy. Emerging evidence suggests the major subtypes—adenocarcinoma (AC) and squamous cell carcinoma (SqCC)—respond differently to therapy. Identification of the molecular differences between these tumor types will have a significant impact in designing novel therapies that can improve the treatment outcome.

**Methods and Findings:**

We used an integrative genomics approach, combing high-resolution comparative genomic hybridization and gene expression microarray profiles, to compare AC and SqCC tumors in order to uncover alterations at the DNA level, with corresponding gene transcription changes, which are selected for during development of lung cancer subtypes. Through the analysis of multiple independent cohorts of clinical tumor samples (>330), normal lung tissues and bronchial epithelial cells obtained by bronchial brushing in smokers without lung cancer, we identified the overexpression of *BRF2*, a gene on Chromosome 8p12, which is specific for development of SqCC of lung. Genetic activation of *BRF2*, which encodes a RNA polymerase III (Pol III) transcription initiation factor, was found to be associated with increased expression of small nuclear RNAs (snRNAs) that are involved in processes essential for cell growth, such as RNA splicing. Ectopic expression of *BRF2* in human bronchial epithelial cells induced a transformed phenotype and demonstrates downstream oncogenic effects, whereas RNA interference (RNAi)-mediated knockdown suppressed growth and colony formation of SqCC cells overexpressing *BRF2*, but not AC cells. Frequent activation of *BRF2* in >35% preinvasive bronchial carcinoma in situ, as well as in dysplastic lesions, provides evidence that *BRF2* expression is an early event in cancer development of this cell lineage.

**Conclusions:**

This is the first study, to our knowledge, to show that the focal amplification of a gene in Chromosome 8p12, plays a key role in squamous cell lineage specificity of the disease. Our data suggest that genetic activation of *BRF2* represents a unique mechanism of SqCC lung tumorigenesis through the increase of Pol III-mediated transcription. It can serve as a marker for lung SqCC and may provide a novel target for therapy.

*Please see later in the article for the Editors' Summary*

## Abstract 2

William Lockwood and colleagues show that the focal amplification of a gene, *BRF2*, on Chromosome 8p12 plays a key role in squamous cell carcinoma of the lung.

## Editors' Summary

**Background:**

Lung cancer is the commonest cause of cancer-related death. Every year, 1.3 million people die from this disease, which is mainly caused by smoking. Most cases of lung cancer are “non-small cell lung cancers” (NSCLCs). Like all cancers, NSCLC starts when cells begin to divide uncontrollably and to move round the body (metastasize) because of changes (mutations) in their genes. These mutations are often in “oncogenes,” genes that, when activated, encourage cell division. Oncogenes can be activated by mutations that alter the properties of the proteins they encode or by mutations that increase the amount of protein made from them, such as gene amplification (an increase in the number of copies of a gene). If NSCLC is diagnosed before it has spread from the lungs (stage I disease), it can be surgically removed and many patients with stage I NSCLC survive for more than 5 years after their diagnosis. Unfortunately, in more than half of patients, NSCLC has metastasized before it is diagnosed. This stage IV NSCLC can be treated with chemotherapy (toxic chemicals that kill fast-growing cancer cells) but only 2% of patients with stage IV lung cancer are alive 5 years after diagnosis.

**Why Was This Study Done?:**

Traditionally, NSCLC has been regarded as a single disease in terms of treatment. However, emerging evidence suggests that the two major subtypes of NSCLC—adenocarcinoma and squamous cell carcinoma (SqCC)—respond differently to chemotherapy. Adenocarcinoma and SqCC start in different types of lung cell and experts think that for each cell type in the body, specific combinations of mutations interact with the cell type's own unique characteristics to provide the growth and survival advantage needed for cancer development. If this is true, then identifying the molecular differences between adenocarcinoma and SqCC could provide targets for more effective therapies for these major subtypes of NSCLC. Amplification of a chromosome region called 8p12 is very common in NSCLC, which suggests that an oncogene that drives lung cancer development is present in this chromosome region. In this study, the researchers investigate this possibility by looking for an amplified gene in the 8p12 chromosome region that makes increased amounts of protein in lung SqCC but not in lung adenocarcinoma.

**What Did the Researchers Do and Find?:**

The researchers used a technique called comparative genomic hybridization to show that focal regions of Chromosome 8p are amplified in about 40% of lung SqCCs, but that DNA loss in this region is the most common alteration in lung adenocarcinomas. Ten genes in the 8p12 chromosome region were expressed at higher levels in the SqCC samples that they examined than in adenocarcinoma samples, they report, and overexpression of five of these genes correlated with amplification of the 8p12 region in the SqCC samples. Only one of the genes—*BRF2*—was more highly expressed in squamous carcinoma cells than in normal bronchial epithelial cells (the cell type that lines the tubes that take air into the lungs and from which SqCC develops). Artificially induced expression of *BRF2* in bronchial epithelial cells made these normal cells behave like tumor cells, whereas reduction of *BRF2* expression in squamous carcinoma cells made them behave more like normal bronchial epithelial cells. Finally, *BRF2* was frequently activated in two early stages of squamous cell carcinoma—bronchial carcinoma *in situ* and dysplastic lesions.

**What Do These Findings Mean?:**

Together, these findings show that the focal amplification of chromosome region 8p12 plays a role in the development of lung SqCC but not in the development of lung adenocarcinoma, the other major subtype of NSCLC. These findings identify *BRF2* (which encodes a RNA polymerase III transcription initiation factor, a protein that is required for the synthesis of RNA molecules that help to control cell growth) as a lung SqCC-specific oncogene and uncover a unique mechanism for lung SqCC development. Most importantly, these findings suggest that genetic activation of *BRF2* could be used as a marker for lung SqCC, which might facilitate the early detection of this type of NSCLC and that BRF2 might provide a new target for therapy.

**Additional Information:**

Please access these Web sites via the online version of this summary at http://dx.doi.org/10.1371/journal.pmed.1000315.

- The US National Cancer Institute provides detailed information for patients and professionals about all aspects of lung cancer, including information on non-small cell carcinoma (in English and Spanish)
- Cancer Research UK also provides information about lung cancer and information on how cancer starts
- MedlinePlus has links to other resources about lung cancer (in English and Spanish)

## Introduction

Lung cancer is the most common cause of cancer deaths worldwide [1],[2]. It is projected that by 2020, lung cancer will be the fifth most lethal entity among all diseases [3]. Improvement in survival has been very modest. Less than 16% of lung cancer patients survive 5 y or more [2], owing to late diagnosis and a paucity of effective therapies.

Squamous cell carcinoma (SqCC) and adenocarcinoma (AC) are the predominant non-small cell lung cancer (NSCLC) cell types [4]. Currently, they are regarded as a single disease entity in terms of systemic therapy. There is increasing evidence that AC and SqCC respond differently to therapy [5],[6]. The differences in therapeutic response may be related to the specific cell lineages from which they develop. Biological differences that segregate with lineage may also lead to differences in progression and response to therapies [7]. Specific genes and their respective pathways may lead to carcinogenesis only when disrupted in permissive conditions [8]. For example, a gene may have oncogenic properties when overexpressed in basal cells in the central airway compartment because it supports growth under these conditions; however, the same gene may have no effect on Clara cells in the lung periphery. Recent studies using transgeneic mouse models support this theory. For example, in murine models with *KRAS* mutations [9]–[12], although all airway epithelial cells contained this mutation, only adenomatous hyperplastic lesions—precursors to AC—developed in the peripheral lung in these mice, suggesting that only particular gene alterations in specific cell types in a certain local environment or niche can lead to the development of the individual lung cancer subtypes.

Cell lineage may also have a dramatic effect on the manifestation of genetic alterations during the development of lung cancer subtypes, as only those promoting a specific malignant phenotype will be selected and maintained [7],[13]. DNA amplification and subsequent overexpression is a predominant mechanism of oncogene activation in epithelial cancers, including those of lung origin [14],[15]. The subsistence of a DNA amplicon is thought to result from selection of genes within the amplified region that promote tumor growth [14]. Thus, the specific requirements for tumorigenesis in different cell lineages may therefore be associated with selection of different amplicons. Copy number increase of 8p12-8p11.21 is one of the most frequent focal changes in NSCLC occurring in ∼9%–35% of cases, with amplification present in ∼3%–8% of cases in the literature, a frequency rivaling those of established NSCLC oncogenes such as *MYC* (∼6%) and *EGFR* (∼3%) [16],[17]. In this study, we sought to determine the lineage specificity of the 8p amplicon in order to discover novel oncogene(s) restricted to tumorigenesis, in particular NSCLC cell types.

## Methods

### Ethics Statement

All patient samples were collected under informed patient consent and anonymized as approved by the University of British Columbia - British Columbia Cancer Agency Research Ethics Board (REB number H04-60060).

### Tumor Samples

Formalin-fixed, paraffin-embedded, and fresh-frozen tissues were collected from St. Paul's Hospital, Vancouver General Hospital and Princess Margaret Hospital following approval by the Research Ethics Boards. Formalin-fixed paraffin-embedded lung carcinoma in situ (CIS) samples were collected by fluorescence bronchoscopy-directed biopsies at the British Columbia Cancer Agency. Hematoxylin and eosin-stained sections of each sample were examined by a lung pathologist to select regions of interest for microdissection to ensure >70% tumor cell content. DNA was isolated using standard procedure with proteinase K digestion followed by phenol-chloroform extraction as previously described [18].

### Tiling-Path Array Comparative Genomic Hybridization

Array hybridization was performed as previously described [19]–[21]. Briefly, equal amounts (200–400 ng) of sample and single male reference genomic DNA were differentially labeled and hybridized to SMRT array v.2 (BCCRC Array Laboratory, Vancouver, BC) previously described to give optimal genome coverage [22],[23].

Hybridized arrays were imaged using a charge-coupled device (CCD) camera system and analyzed using SoftWoRx Tracker Spot Analysis software (Applied Precision). Systematic biases (intensity, spatial, plate, and background biases) were removed from all array data files using a stepwise normalization procedure as previously described [15],[24]. SeeGH software was used to combine replicates and visualize all data as log_2_ ratio plots [25],[26]. All replicate spots with a standard deviation above 0.075 or signal-to-noise ratios below three were removed from further analysis. The clones were then positioned on the basis of the human March 2006 (hg18) genome assembly. Genomic imbalances (gains and losses) within each sample were identified using aCGH-Smooth [27] with lambda and breakpoint per chromosome settings at 6.75 and 100, respectively, as previously described [21]. The resulting frequency of alteration was then determined for each lung cancer cell type as described previously [21]. High-level amplifications were determined using an algorithm previously described with the log_2_ threshold set at >0.6 for tumors and >0.8 for CIS cases (because of different levels of cell heterogeneity) [15]. Regions were only scored as amplified if two or more consecutive array elements met these criteria. All array comparative genomic hybridization (CGH) data are available on the System for Integrative Genomic Microarray Analysis (SIGMA) Web site at http://sigma.bccrc.ca/.

### Comparison of Cell Type Alteration Frequencies

Regions of differential copy number alteration between AC and SqCC genomes were identified as follows. Each array element was scored as 1, gain/amplification; 0, neutral/retention; or −1, loss/deleted, for each individual sample. Values for elements filtered on the basis of quality control criteria were inferred by using neighbouring clones within 10 Mb. Probes were then aggregated into genomic regions if the similarity in copy number status between adjacent clones was at least 90% across all samples from the same cell type. The occurrence of copy number gain/amplification, loss/deletion, and retention at each locus was then compared between AC and SqCC datasets using the Fisher exact test. Testing was performed using the *R* statistical computing environment on a 3×2 contingency table as previously described, generating a *p*-value for each clone [21]. A Benjamini-Hochberg multiple hypothesis testing correction based on the number of distinct regions was applied and resulting *p*-values ≤0.01 were considered significant. Adjacent regions within 1 Mb that matched both the direction of copy number difference and statistical significance were then merged. Finally, regions had to be altered in >20% of samples in a group and the difference between groups >10% to be considered.

### Gene Expression Microarray Analysis of Clinical Tumor Specimens

47 fresh-frozen lung tumors were obtained from Vancouver General Hospital as described above. Microdissection of tumor cells was performed and total RNA was isolated using RNeasy Mini kits (QIAGEN Inc.). Samples along with universal reference RNA were labeled and hybridized to a custom Agilent Whole Genome Oligonucleotide microarray according to the manufacture's protocols. The resulting expression data were processed and normalized using Rosetta Resolver software. Affymetrix U133 Plus 2 expression data for 111 NSCLC tumors were downloaded from the Gene Expression Omnibus (http://www.ncbi.nlm.nih.gov/geo/, accession number GSE3141) and normalized using Microarray Suite (MAS) 5.0 [28]. A summary containing the number of samples analyzed and corresponding platform is presented in Table S1.

### Gene Expression Microarray Analysis of Normal Bronchial Epithelial Cells

RNA was obtained from exfoliated bronchial cells of 67 lung cancer–free individuals obtained during fluorescence bronchoscopy [29]. All individuals were either current or former smokers without lung cancer. Expression profiles were generated for all cases using the Affymetrix U133 Plus 2 platform and normalized using MAS 5.0.

### Statistical Analysis of Gene Expression Data

Gene expression probes were mapped to March 2006 (hg18) genomic coordinates and those within the regions of copy number difference between the cell types on Chromosome arm 8p were determined. Comparisons between expression levels for AC and SqCC tumors as well as SqCC tumors and normal bronchial cells were performed using the Mann-Whitney U test and computed with the ranksum function in MATLAB. As the direction of gene expression difference was predicted to match the direction of copy number difference, one-tailed *p*-values were calculated. A Benjamini-Hochberg multiple hypothesis testing correction was applied on the basis of the total number of gene expression probes analyzed. Probes with a corrected *p*-value ≤0.01 were considered significant. If multiple probes mapped to the same gene, the one with the lowest *p*-value (Agilent data) or with maximum intensity across the data (Affymetrix) was used.

### Integration of Genetic and Gene Expression Data

To integrate gene expression with copy number data, two methods were used. First, a 10-kb moving average was generated using the normalized log_2_ array CGH ratios for each sample with copy number and expression. These values were subsequently standardized using a Z-transformation for each sample throughout the whole genome in order to facilitate better comparisons across the sample set. An average Z-score was then calculated using the values corresponding to the genomic intervals spanning each of the genes of interest on Chromosome 8p. Finally, a nonparametric Spearman correlation coefficient was calculated using the Z-scores for copy number and log_10_ ratios for gene expression across all samples of interest. The corresponding *p*-value representing the statistical significance of a positive correlation was calculated and a Benjamini-Hochberg multiple hypothesis testing correction applied as described above. For the second method, the copy number status was determined by aCGH-Smooth as described above and mapped to genes of interest from clones using genomic coordinates from the UCSC Genome Browser (hg18). The gene expression levels for all genes were then compared between samples with copy number gain/amplification against samples that were copy number neutral using the Mann-Whitney U test [15]. An association was deemed significant if the Benjamini-Hochberg corrected *p*-value ≤0.05 and the median and mean gene expression in the samples with gain/amplification were higher than those samples that were copy number neutral. Again, as the direction of gene expression difference was predicted to match the direction of copy number difference, one-tailed *p*-values were calculated.

### Reverse Transcriptase PCR Analysis of Transcription Levels in Clinical Tumor Samples

Quantitative reverse transcriptase (RT)-PCR was performed on SDS7900HT (Applied Biosystems) using SYBR Green and the ΔΔCt method with *RPS13* expression levels used as reference for normalization. Primers used were: BRF2_F: GTGAAGCTCCTGGGACTGGAT, BRF2_R: GTATTTGGCTGGCACAGAAGG, RPS13F: GTTGCTGTTCGAAAGCATCTTG, and RPS13R: AATATCGAGCCAAACGGTGAA. Associations between *BRF2* expression and clinicopathological features were evaluated by the Wilcoxon test. Breakdown of samples used are provided in Table S1.

### Cell Lines and Culture Conditions

NSCLC cell lines H520, H1395, and H2347 were purchased from American Type Culture Collection (ATCC). Cells were maintained in RPMI-1640 medium (Invitrogen) supplemented with 10% fetal bovine serum (Invitrogen). The HBEC3-KT immortalized normal human bronchial epithelial cell (HBEC) line was established by introducing mouse Cdk4 and hTERT into normal HBECs obtained from a 65-y-old woman without cancer [30]. The HBEC3-KT53 line was established by stably knocking down p53 in the original cell line, HBEC3-KT [31]. These two parental lines were used to overexpress BRF2 via the pMSCV vector (see below). All HBEC3 cell lines were cultured in K-SFM (Invitrogen) medium containing 50 µg/µl bovine pituitary extract (Invitrogen) and 5 ng/µl EGF (Invitrogen).

### TaqMan Analysis of Transcript Levels in Cancer Cell Lines

5 µg micrograms of total RNA isolated from cultured cells (H2122, H2347, HCC193, H1395, H2009, H1993, HCC4006, HCC2279, H2087, HCC78, HCC461, HCC1195, H1819, H1648, HCC366, H3255, HCC2450, HCC15, HCC95, and H520) was converted to cDNA using an ABI High Capacity cDNA Archive kit (Applied Biosystems). An aliquot of 100 ng of cDNA was used for each real-time PCR reaction. TaqMan (Applied Biosystems) gene expression assays: BRF2 (Hs00217757_m1) and 18s rRNA (Hs99999901_s1) were performed using standard TaqMan reagents and protocols on a Applied Biosystems 7500 Fast Real-Time PCR system (Applied Biosystems). The **ΔΔ**Ct method was used for expression quantification using the average cycle threshold for 18S rRNA to normalize gene expression levels between samples [21]. Cycle thresholds for the primers were then compared between the individual cell lines and a pooled normal lung cDNA reference sample generated from Human Lung Total RNA (AM7968, Ambion) to identify the fold change represented.

### Western Blot Analysis of Protein Levels

Cells were washed twice with cold PBS and lysed in the presence of protease inhibitors. Each cleared lysate was diluted and boiled for electrophoresis and transferred to polyvinylidene membrane [31]. Membranes preblocked with 3% bovine serum albumin in PBS with 0.05% Tween-20 (PBST) were incubated with primary antibodies against BRF2 (Abcam, 1∶500 dilution) for 1 h at room temperature. After three washes in PBST, the membranes were incubated with horseradish-peroxidase-conjugated donkey anti-goat polyclonal antibody (Abcam, 1∶2,000 dilution) for 45 min at room temperature. After three PBST washes, antibody binding was visualized by enhanced chemiluminescence (GE Healthcare). Subsequently, the bound antibodies were stripped from the membranes with a buffer containing 62.5 mM Tris-HCl, (pH 6.7), 2% SDS and β-mercaptoethanol and reprobed with monoclonal antibody to beta-actin (Abcam, 1∶6,000) to confirm equal sample loading.

### Northern Blot Analysis of Pol III Transcript Levels

Briefly, total RNA was isolated from cell lines using TRIzol reagent (Invitrogen) and 4 µg was resolved on an 8% UreaGel denaturing gradient gel as per manufacturer's instructions (National Diagnostics). The gel was then equilibrated in 0.5× TBE for 15 min and RNA was capillary transferred to positively charged nylon membrane using 0.5× TBE according to the protocol described by Sambrook and Russell [32]. RNA was bound to the membrane by UV-crosslinking with 120 J UV light using a Stratalinker (Stratagene). The blot was then prehybridized at 45°C for 30 min using DIG Easy hybridization buffer (Roche), and 50 pmol of DIG labeled30-mer oligonucleotide probe was then added. Specific probe sequences were as follows: 5S rRNA, 5′- CCTGCTTAGCTTCCGAGATCAGACGAGATC-3′; U6 small nuclear RNA (snRNA), 5′- CTTGCGCAGGGGCCATGCTAATCTTCTCTG-3′; 7SK snRNA, 5′-CGTCCTCTTCGACCGAGCGCGCAGCTTCGG-3′. Hybridization proceeded for 16–20 h and blots were washed two times for 15 min at room temperature in 2× SSC, 1% SDS followed by a 15-min wash in the same buffer at 45°C. Further washes and luminescence detection was performed using the DIG Wash and Block Buffer set and the DIG Luminescent Detection kit following the manufacturer's instructions (Roche). Blots were then exposed to Lumi-Film (Roche) and developed to image.

### RNAi Knockdown

For RNA interference (RNAi) experiments, two methods were employed. For the first method, lentiviral short hairpin RNAs (shRNA) vectors targeted against *BRF2* were purchased from Open Biosystems. Briefly, individual lentiviruses, each containing a single pLKO plasmid construct coding an shRNA targeted for *BRF2*, were prepared by transfecting 293T cells with the packaging plasmids VSVG and d8.91 and the shRNA plasmids using TransIT-LT1 transfection reagent (Mirus). Virus containing empty pLKO vector served as a control. Virus supernatant was collected from the transfected 293T cells each day for 3 consecutive days post-transfection. H520 cells were infected at 50% confluency, using 1 ml of each respective virus. After 48 h, cells were selected with 2.5 µg/ml puromycin. Selection was continued until all nontransfected cells were dead. Stably transfected cell lines were maintained in growth media supplemented with 2.5 µg/ml of puromycin. Sequence details for *BRF2*-1 (TRCN0000016128) and *BRF2*-2 (TRCN0000016129) can be found on the Open Biosystems Web site (www.openbiosystems.com).

For the second method, ON-TARGETplus SMARTpool small interfering RNAs (siRNAs) targeting BRF2 along with a negative control (ON-TARGETplus siCONTROL nontargeting siRNA pool) were obtained from Dharmacon. H520 and H1395 cells were subcultured at a ratio of 1∶3 or 1∶6 using 0.25% tryspin-EDTA (Gibco). Transfection efficiency was optimized using siGLO Green Transfection Indicator (Dharmacon). For the transfection experiments, cells were seeded in 24-well culture plates at 60,000 cells/ml 24 h before transfection. Cells were transfected at a final concentration of 100 nM siRNA using Lipofectamine RNAiMAX (Invitrogen) according to the manufacturer's instructions. The cells were then incubated at 37°C for 24 h before RNA analysis, 48 h for protein, and 72 h for MTT assays. For both shRNA and siRNA experiments, *BRF2* expression levels for multiple independent knockdowns were determined by qRT-PCR as described above and scaled relative to the average of the control treated cells (±standard error measure [SEM]).

### Cell Proliferation Assays

The 3-[4, 5-dimethylthiazol-2-yl]-2, 5-diphenyltetrazolium bromide (MTT) assay (Trevigen) was used to determine the status of cell proliferation in both shRNA and siRNA experiments according to the manufacturer's instructions. For siRNA experiments, exponentially growing cells were diluted to a concentration of 313,000 cells/ml in RPMI-1640 with 10% FBS, seeded in triplicate in 96-well plates and incubated at 37°C for 4 h. The cells were then treated with 10 µl of MTT reagent for 4 h before adding 100 µl of detergent reagent to solubilize the formazan precipitate. The reaction product was then quantified by measuring absorbance at 570 nm with reference to 650 nm using an EMax plate reader (Molecular Devices). The mean ±SEM absorbance values for experiments from three independent transfections were normalized to the average of the respective controls.

For shRNA experiments, 4,000 cells for each condition were seeded in triplicate for each time point in 96-well plates and treated with MTT as described above at 24, 72, and 96 h. The absorbance reading for blank (media) wells was subtracted from the mean absorbance readings for each time point and plotted (±SEM) to quantify cell proliferation. Replicate experiments were performed and a representative experiment is shown.

### Soft Agar Anchorage-Independent Growth Assay

The H520 stably transfected pLKO vector control and shRNA *BRF2* gene knockdown cell lines were used in the colony formation assay. Single cell suspensions were prepared in growth media supplemented with 20% fetal bovine serum (Invitrogen), 2.5 µg/ml puromycin, 0.1% penicillin-streptomycin (Invitrogen), and 0.3% low-melting point agarose (Invitrogen), resulting in a final concentration of 1,000 cells/ml. 1 ml of cell suspension (1,000 cells) was plated onto an equal volume of supplemented media with a 0.5% low-melting point agarose concentration. Supplemented media lacking cells was plated as a negative control. Each cell line was seeded in triplicate in 12-well plates and cultured for 14 d at 37°C, after which colonies were counted and the mean ±SEM were normalized to the average of the control. Replicate experiments were performed and a representative experiment is shown.

### Construction of the BRF2 Expression Vector

The BRF2 sequence from the pBRF2-HORF construct (Invitrogen) was cloned into the retroviral vector pMSCV-hygro (Clontech) and sequenced. This construct was named pMSCV-BRF2. The pMSCV-BRF2 construct and the vector alone (pMSCV) were then transfected into the Pheonix Ampho retroviral packaging cell line (Orbigen) according to manufacturer's protocols. Subsequent infections into HBEC3-KT and HBEC3-KT53 were performed and plasmid-containing cells were selected by treating with 20 µg/µl of hygromycin for 10 d. This step resulted in the generation of four stable cell lines: HBEC3-KT-pMSCV-BRF2, HBEC3-KT-pMSCV (vector control), HBEC3-KT53-pMSCV-BRF2, and HBEC3-KT53-pMSCV (vector control).

### In Vitro Cell Growth Assays

Growth curves were determined for each of the six HBEC cell lines by culturing 1,000 cells in triplicate in 12-well plates and counting on the third, sixth, eighth, and tenth day. The average ±SEM for each line is reported. Replicate experiments were performed and a representative experiment is shown.

### Statistical Analysis of Functional Assays

For all cell model assays, *p*-values were calculated using the Student's *t* test when comparing two conditions and ANOVA when comparing three. All calculations were performed with MATLAB software and one-tailed *p*-value ≤0.05 was considered significant.

### Immunohistochemistry

Slides were deparaffinized using xylene and rehydrated through an ethanol series to water. Antigen retrieval was performed using a decloaking chamber at 15 psi for 20 min in sodium citrate buffer (pH 6.0). Endogenous peroxidase enzyme activity was blocked using 3% H2O2 in methanol for 30 min at room temperature. Slides were washed in 1% PBS and then blocked using 10% skim milk for 6 h at room temperature. Slides were incubated for 16 h at 4°C with a 1∶200 dilution of goat polyclonal anti-BRF2 primary antibody (Abcam), followed by incubation with a donkey anti-goat biotinylated secondary antibody (Santa Cruz Biotechnology). Normal goat IgG was used as negative control (Santa Cruz Biotechnology). Detection was accomplished using DAB (ImmunoCruz staining system, Santa Cruz Biotechnology). Slides were then counterstained using hematoxylin, and the area within the diagnostic area was scored by three independent observers on the basis of the following criteria: 0, no positive staining; 1, 25% positive cells; 2, 50% positive cells; 3, 75% positive cells; and 4, 100% positive cells. Conflicting scores were resolved by choosing the value consistent between two observers or the average of all three varying scores.

### Significance Analysis of Microarrays

Using the 111 NSCLC samples in the dataset by Bild et al. (Table S1) [28], samples were sorted by highest to lowest expression for BRF2 on the basis of the probe with the highest average intensity across the dataset [28]. Differential gene expression analysis using significance analysis of microarrays (SAM) [33] was performed using the ten samples with highest BRF2 expression against the ten samples with lowest *BRF2* expression. A *q*-value threshold of ≤0.05 was used to identify differentially expressed genes associated with high BRF2 expression.

### Functional Assessment of BRF2-Associated Genes

Functional analyses were generated through the use of Ingenuity Pathways Analysis (Ingenuity Systems) as previously described [15]. Functional Analysis identified the biological functions that were most significant to the dataset. Fisher exact test was used to calculate a *p*-value determining the probability that each biological function assigned to the dataset is due to chance alone.

## Results

### A Focal Region on 8p Is Preferentially Amplified in SqCC

**Figure 1:**
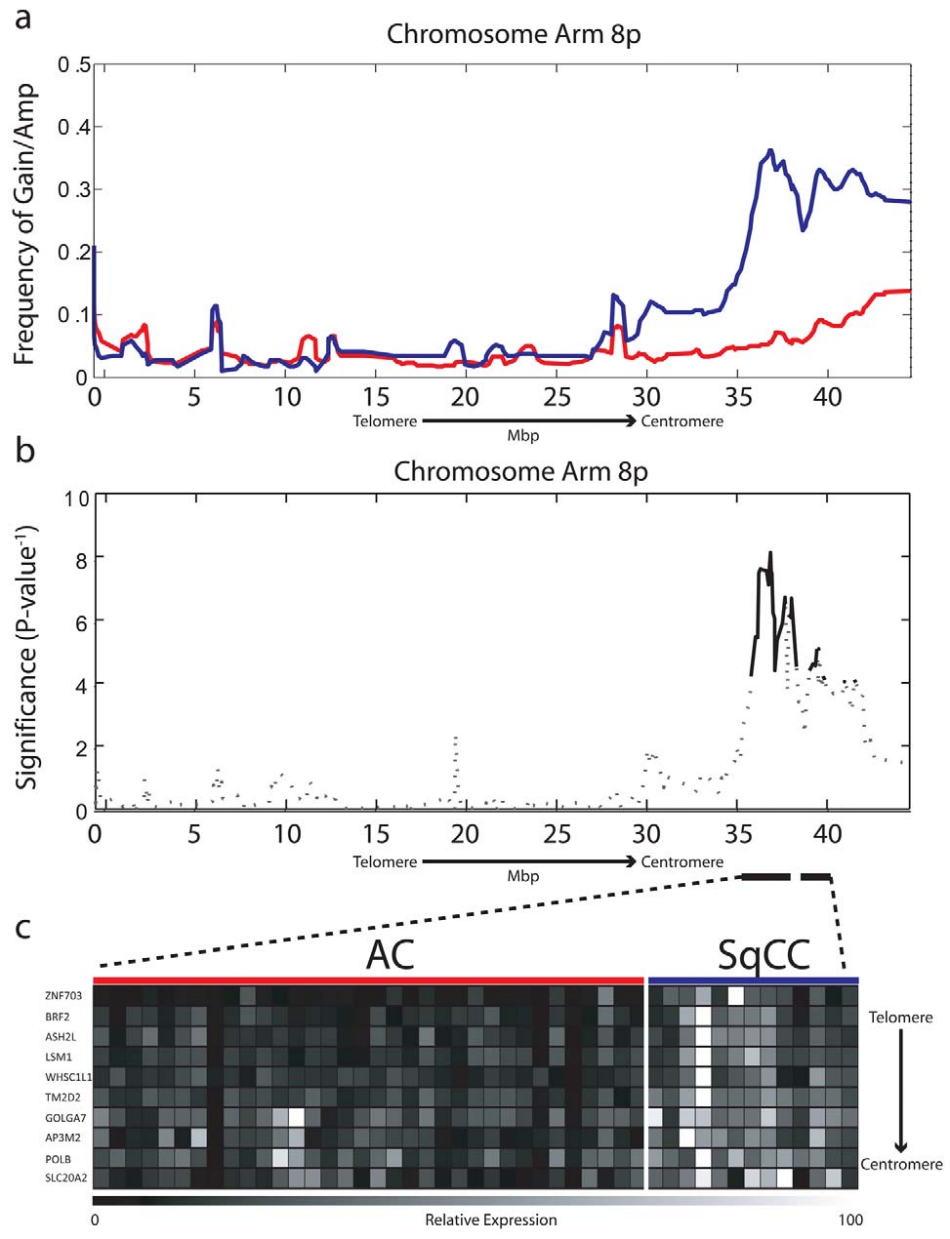
Chromosome 8p amplification in NSCLC is restricted to the SqCC lineage. (A) Frequency of gain/amplification along Chromosome arm 8p is depicted for 103 AC (red) and 58 SqCC clinical tumor specimens (blue). (B) The significance of copy number disparity (inverse *p*-value) between AC and SqCC cell type groups is depicted for 8p. Solid black lines represent regions considered statistically different (*p*≤0.01), whereas dashed lines are not. (C) Relative expression for genes within regions of copy number difference, which were also expressed at significantly higher levels in SqCC (*n* = 13) compared to AC (*n* = 34) tumors (*p*≤0.01). The color scale ranges from black (low expression) to white (high expression).

We compared the 8p chromosome arm of 161 microdissected NSCLC tumors—103 AC and 58 SqCC (Table S1, sample set 1a)—by tiling resolution array CGH [22]. After hybridization experiments, genomic profiles were normalized and subjected to a smoothing algorithm in order to computationally define regions of copy number gain and loss along the entire length of Chromosome arm 8p [21]. Individual samples were then grouped by their corresponding cell type, and probes were aggregated into regions on the basis of similar copy number status. The resulting frequency of alteration for each region along the arm was compared between cell types using the Fisher exact test to identify regions of copy number disparity, and the resulting *p*-values were corrected for multiple comparisons with a cut-off of ≤0.01 considered significant (Methods). Although the telomeric portion of 8p was frequently lost in both AC and SqCC cell types, two regions spanning a total of 5.65 Mbp at 8p12–8p11.21 were found to be frequently gained specifically in SqCC (Figure 1a and 1b; Table S2). Copy number increase of focal regions at 8p12–p11.21 was found in up to 40% of SqCC tumors, while DNA loss was the most prevalent event in AC (∼39%). In addition, high-level amplification (log_2_ ratio >0.6) was present in ∼12% of SqCC samples (seven out of 58) demonstrating the preferential selection for this alteration in tumors of this cell lineage. The increased incidence of 8p amplification in comparison to previous reports is attributed to analyzing the cell types as distinct groups, as opposed to combining all the NSCLC cell types as a single entity. In addition, the small sample sizes of previous studies may also have limited the detection of specific disruptions unique to each cell type to just extremely high frequency events, such as the gain of Chromosome 3q [17],[34],[35]. These results indicate that gain/amplification of 8p12–8p11.21 is restricted to SqCC and occurs far more frequently than previously thought, highlighting the importance of considering cell lineage in genomic studies of malignancies from the same tissue site.

### BRF2 Gene Expression Drives Selection of the 8p Amplicon in Lung SqCC

The cell type dependent pattern of 8p amplification raised the possibility that a lineage-specific oncogene may be driving the preferential selection of this amplicon in SqCC. Such a gene should display five fundamental properties each translating into its own testable hypothesis. First, increased expression would be restricted to SqCC tumors mirroring the specificity of DNA amplification (hypothesis 1). Second, as the target of the amplicon, expression would be higher in SqCC tumors with gain/amplification than those without (hypothesis 2). Third, expression should be significantly higher in SqCC tumors than normal bronchial epithelial cells; that is, the gene should be activated in cancerous and not normal tissue (hypothesis 3). Fourth, the gene should have oncogenic potential and provide a growth and/or survival advantage to cells when overexpressed (hypothesis 4). Lastly, if necessary for initiating tumorigenesis, amplification should occur early in tumor development and therefore be present in lung SqCC precursor lesions (hypothesis 5).

To test the first hypothesis, we generated gene expression microarray profiles for a subset of 47 tumors (34 AC, 13 SqCC) with sufficient amounts of material that were also analyzed by array CGH in order to integrate genetic and gene expression information (Table S1, sample set 1b). In total, 62 probes corresponding to 44 unique genes mapped to within the alteration boundaries (Table S2). To identify lineage-restricted genes, we compared the expression levels for all probes between the AC and SqCC samples. Since we predicted candidate genes to be overexpressed in SqCC, a one-tailed Mann-Whitney U test was used with Benjamini-Hochberg corrected *p*-values ≤0.01 considered significant. Ten unique genes meeting these criteria were uncovered from this analysis that showed a clear distinction in expression levels between the AC and SqCC tumors (Figure 1c; Table S3).

After identifying these SqCC specific genes, we next aimed to ensure that amplification is responsible for their differential expression, as these will be candidate targets driving amplicon selection (hypothesis 2). For this purpose, we utilized two complementary approaches. First, a nonparametric Spearman correlation coefficient was calculated for each gene using Z-transformed copy number ratios and log_10_ gene expression ratios (Methods). Five of the ten genes (*LSM1*, *BRF2*, *ASH2L*, *TM2D2*, and *WHSC1L1*) had a correlation coefficient of >0.75 and a corrected *p*-value (representing the statistical significance of a positive correlation) of <0.01 and were further considered as candidates (Table S3 for all values). The second approach involved the comparison of expression levels between SqCC tumors with gene dosage increase (gain/amplification) and those with neutral copy number status (Methods). Of the five genes with a positive association between copy number and expression, only three (*LSM1*, *BRF2*, and *ASH2L*) also showed significantly elevated transcript levels specifically in SqCC samples with gain or amplification and were therefore determined to be regulated by copy number (Table S3). qRT-PCR analysis of *BRF2* (the most likely target gene, see below) confirmed the microarray results (Figure S1; Table S4). Importantly, none of these genes demonstrated a correlation between copy number and expression in AC, reinforcing the specificity of this alteration to SqCC.

**Figure 2:**
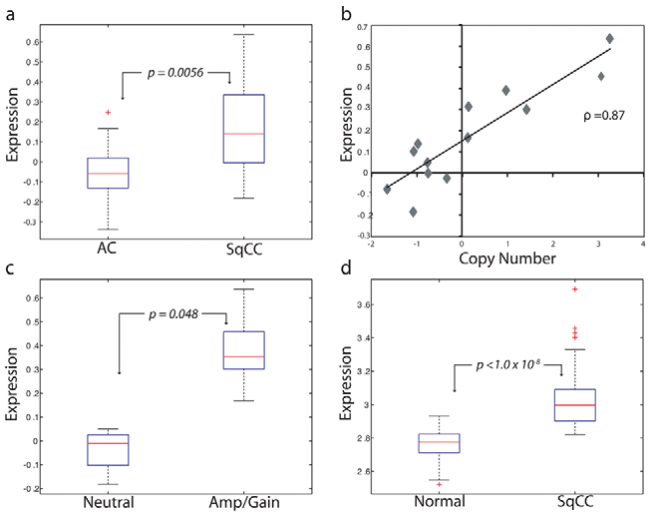
*BRF2* is a lineage-specific oncogene targeted by amplification in SqCC. (A) Comparison of *BRF2* mRNA expression values for AC (*n* = 34) and SqCC (*n* = 13) tumors (*p* = 0.0056). Box-plots depict the median group expression (red line), the 25th and 75th percentiles (blue box), and the limits of 95% of samples for each group (outside lines) with values for all other samples represented by red crosses. Expression values for all plots are in arbitrary log10 units. (B) Spearman's correlation of Z-transformed array CGH copy number ratios and expression values for *BRF2* in 13 SqCC tumors (correlation coefficient = 0.87). Each diamond represents an individual sample. (C) Comparison of *BRF2* expression between SqCC tumors with neutral copy number status (*n* = 4) and SqCC tumors with gain/amplification (*n* = 6) (*p* = 0.048). (D) Difference in *BRF2* expression levels between 67 exfoliated bronchial cell samples from cancer-free patients and 53 SqCC tumors from an independent sample set (*p*<1.0×10^−8^).

In addition to demonstrating a linkage between expression and amplification, a candidate oncogene should only be expressed at elevated levels in cancerous, and not normal, tissues [36]. Therefore, to test the third hypothesis, we analyzed the RNA levels of these three genes in an independent panel of 53 SqCC lung tumors and 67 samples of exfoliated bronchial cells from cancer-free individuals generated using the Affymetrix U133 Plus 2 platform (Table S1, sample sets 2 and 3). Strikingly, only *BRF2* was aberrantly expressed (>2-fold, *p*<1.0×10^−8^) in cancerous tissues identifying it as the sole gene passing the three main criteria of a candidate lineage-specific oncogene described above (Figure 2; Table S3). To further confirm these observations, a third, independent sample set consisting of 118 NSCLC tumors and 39 non-neoplastic lung tissues (Table S1, sample set 4) was analyzed for *BRF2* expression by qRT-PCR (Methods). Consistent with the microarray results, expression of *BRF2* in primary tumors was significantly higher than that in the non-neoplasia tissues (*p*<0.001) with overexpression more common in SqCC than AC (*p* = 0.03), supporting our findings.

Taken together, results from testing the first three hypotheses clearly demonstrate that *BRF2* is the driver gene of the 8p amplicon and identify it as a candidate lineage-specific oncogene in SqCC. Previous studies investigating this amplified region in NSCLC have proposed *FGFR1* and *WHSC1L1* as potential oncogenes [17],[37]. However, we ruled out *FGFR1* as a possible target as it was not differentially expressed between AC and SqCC, and as such, was excluded from further analysis. This conclusion is in agreement with a study by Tonon et al. that suggested *WHSC1L1* as the more likely amplification target in NSCLC [17]. Although we demonstrated that *WHSC1L1* expression was restricted to SqCC and correlated with increased gene dosage, it was not significantly higher in samples with gain/amplification or different between normal and cancerous cells (*p* = 0.12, fold change = 1.3), and therefore, also discounted.

### BRF2 Contributes to SqCC Tumorigenesis by Regulating Cell Growth and Proliferation through the Increase of Polymerase III Activity

*BRF2* encodes a subunit of a transcription initiation complex responsible for RNA polymerase III (Pol III)-mediated transcription [38],[39]. Pol III transcribes a limited set of genes that encode nontranslated RNAs including 5S rRNA, tRNA, 7SL RNA, and U6 RNA, which are essential for protein synthesis and RNA processing [40]. Because these processes are fundamental determinates of the capacity of a cell to grow, increased activity of Pol III is often observed during cancer development [41]. Indeed, transformed cells express elevated levels of Pol III transcripts, and inhibition of these transcripts limits cell growth and proliferation [42]. It has been proposed that deregulation of Pol III in transformed cells can occur through three different mechanisms: release from cellular repressors, direct activation by oncogenes, and overexpression of transcription factors [40]. In normal cells where growth is tightly controlled, tumor suppressors including RB, p53, and PTEN repress Pol III transcription [43],[44]. Inactivation of these genes or activation of oncogenes such as *MYC* and *ERK* reverse this process [42],[44],[45]. Interestingly, the majority of these genes are mutated in lung cancer, representing a potential mechanism of increasing Pol III activity, and subsequently, cell growth potential during tumorigenesis. Transcription factors, however, are often the limiting components of Pol III-mediated transcription and elevated levels of these components have been observed in numerous cancer types [41]. Recently, the overexpression of another Pol III transcription factor *BRF1* has been shown to increase Pol III-mediated transcription, resulting in the transformation of cells *in vitro* and tumor formation *in vivo* [46],[47]. A study by Marshall et al. was the first to implicate Pol III deregulation as a causative factor in cancer formation [46]; however, no studies have been reported to date of activating mutations in Pol III subunits or associated transcription factors in tumors. Therefore, we hypothesized that the amplification and overexpression of *BRF2* may contribute to lung SqCC tumorigenesis by contributing to increased cell growth and proliferation, representing a novel alternative mechanism of increasing Pol III transcription in cancer.

**Figure 3:**
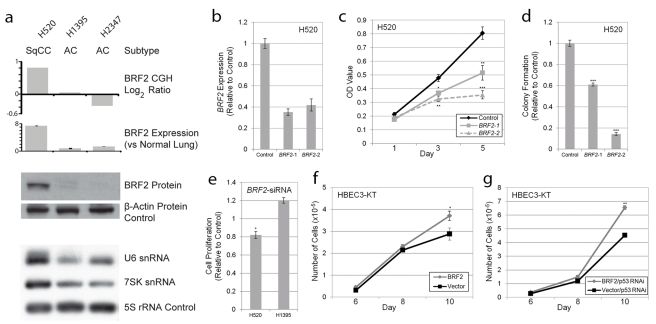
BRF2 activation contributes to cell growth and proliferation. (A) Concordance between *BRF2* copy number (array CGH), expression (qRT-PCR), protein (immunoblot) levels, and Pol III transcript levels (northern blot) in H520, H1395, and H2347 NSCLC cell lines. (B) Decrease in *BRF2* mRNA levels in H520 cells expressing shRNAs targeting *BRF2* relative to those expressing a negative vector control (mean ± SEM of triplicate samples). (C) *BRF2* knockdown results in decreased cell proliferation in H520 cells with amplification and overexpression as measured by MTT assay relative to vector control (mean ± SEM of triplicate samples). (D) *BRF2* knockdown reduces colony growth of H520 cells in soft-agar relative to vector control (mean ± SEM of triplicate samples). (E) siRNA transfection results in decreased cell proliferation as measured by the MTT assay in H520 SqCC cells but not H1395 AC cells relative to nontargeting siRNA control (mean ± SEM of triplicate experiments). Increased saturation density in both (F) BRF2 expressing HBEC and (G) BRF2 and p53RNAi expressing HBEC compared to their respective controls (mean ± SEM of triplicate samples). *, *p*<0.05; **, *p*<0.01; ***, *p*<0.001 (Student's *t* test; compared to control).

To test this hypothesis (hypothesis 4), we performed complementary loss and gain of function in vitro experiments using lung cancer cell lines and immortalized HBEC lines, respectively. Twenty NSCLC cell lines (16 AC and 4 SqCC) previously analyzed by array CGH were assayed for *BRF2* expression by qRT-PCR (Methods). Mirroring the findings from the clinical tumor specimens, *BRF2* expression was strongly correlated with gene dosage with the two cell lines with amplification (HCC95 and H520) displaying the highest transcript levels (Figure S2; Table S5). In addition, both these lines were derived from SqCC samples and no AC cell lines contained amplification, re-enforcing the lineage specificity of *BRF2* activation. To determine the effect of *BRF2* overexpression on BRF2 protein levels, three cell lines were selected for Western blot analysis: a SqCC with amplification (H520), an AC with neutral copy number (H1395), and an AC with loss (H2347) (Figure 3a). Consistent with a role in tumorigenesis, high protein levels were only found in H520.

To determine if increased BRF2 levels lead to higher Pol III activity, we performed Northern blot analysis to assess the expression of Pol III-mediated transcripts. BRF2 is specifically involved in transcription from type 3 (gene external) Pol III promoters, which are responsible for the expression of snRNA genes, two of the best characterized of which are U6 and 7SK [48]–[50]. This finding is in contrast to type 1 and type 2 Pol III promoters (gene internal), which require BRF1 for transcription and are involved in the expression of 5S rRNA and tRNA genes, respectively [48],[50]. Thus, we hypothesized that BRF2 activation would lead to an increase only in type 3 transcripts and not those regulated by BRF1. As expected, assessment of transcript levels in the lung cancer lines showed drastically higher levels of both U6 and 7SK relative to 5S loading control in H520 cells compared to H1395 and H2347 cells, confirming increased BRF2-dependent Pol III transcription upon BRF2 activation (Figure 3a). Furthermore, U6 levels were decreased upon knockdown of *BRF2* expression in H520 cells using an shRNA construct (Figure S3). These results match those of a recent study, which showed that BRF2 protein levels correlate with U6 promoter activity [51]. These data suggest that increased BRF2 levels are sufficient to increase Pol III activity, demonstrating the downstream mechanistic effect of gene amplification.

To assess the functional significance of *BRF2* amplification and overexpression on SqCC development, RNAi-mediated knockdown was performed in H520 cells. Expression of two different shRNAs) targeting *BRF2* substantially reduced transcript levels (Figure 3b) and significantly decreased cell proliferation compared to a negative vector control (Figure 3c). In addition, knockdown of *BRF2* expression significantly reduced the ability of these cells to grow in an anchorage-independent manner as measured by colony formation in soft agar (Figure 3d). Similar results on cell proliferation were observed with *BRF2* siRNA pool transfection of H520 cells (Figure 3e). In contrast, siRNA knockdown of an AC cell line without *BRF2* amplification and overexpression, H1395, did not diminish cell proliferation and resulted in an increase in proliferation relative to transfection with a nontargeting control siRNA pool (Figure 3e). How knockdown of *BRF2* could lead to an increase in cell proliferation in this context remains unclear and warrants further investigation. Lastly, to further confirm the specificity of this effect to cell lines with amplification, we also performed knockdown experiments in two SqCC cell lines (HCC15 and HCC2450) without *BRF2* amplification (Figure S4). As expected, no significant decrease in proliferation was seen in HCC15 or HCC2450 upon BRF2 inhibition. These results demonstrate a crucial role for *BRF2* in contributing to the sustained cellular proliferation and survival of SqCC tumors with gene activation and highlight its cell type specific oncogenic potential in lung cancer.

To further validate its tumorigenic properties, we performed complimentary experiments by overexpressing *BRF2* by stable transduction of immortalized HBEC lines (Figure S5) and measured cell growth compared to vector-expressing controls. HBEC lines are immortalized without the use of viral oncoproteins, have minimal genetic changes, and do not exhibit a transformed phenotype [30],[31]. In addition, since they express epithelial markers and morphology and can differentiate into mature airway cells, they represent an attractive model for testing the importance of specific gene alteration found in the initiation of epithelium-derived lung cancer [30],[31]. Strikingly, the introduction of *BRF2* alone resulted in a modest but significant increase in cellular growth and saturation density, further supporting a tumorigenic role for this gene (Figure 3f). Furthermore, as p53 is inactivated in ∼50% of NSCLC tumors, and is known to repress Pol III-mediated transcription, we sought to investigate the impact of *BRF2* overexpression in conjunction with p53 silencing on HBEC growth. Interestingly, the combination of these two alterations enhanced cell growth greater than each alteration alone (*p* = 2.36×10^−5^), suggesting a synergistic role for these alterations in promoting proliferation (Figure 3g). Taken together, our results demonstrate that *BRF2* overexpression plays a key role in regulating cell growth and proliferation, confirming the functional significance of *BRF2* gene amplification in SqCC.

### BRF2 Activation Is an Early Event in SqCC Development

**Figure 4:**
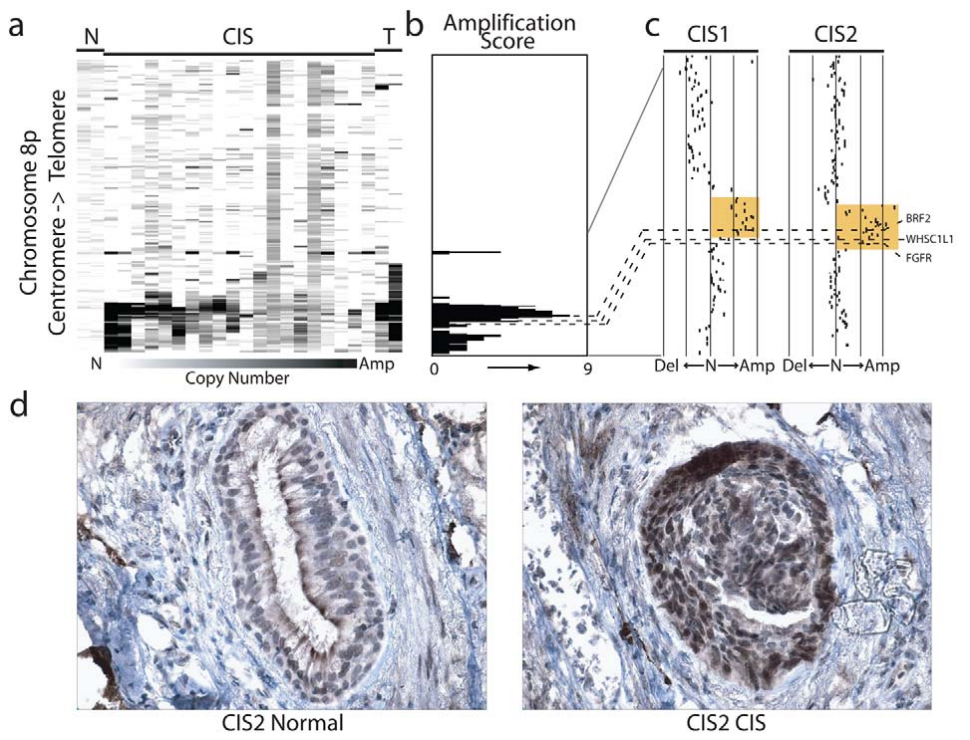
Amplification and overexpression of BRF2 in preinvasive SqCC lesions. (A) Frequent copy number increase of Chromosome arm 8p in 20 bronchial CIS lesions. Samples are ordered in columns and ordered by genomic position along 8p. The color scale ranges from white (neutral copy number, N) to black (amplification, Amp). Data from representative normal lung (N) and SqCC tumor samples (T) are displayed to the left and right of the CIS cases respectively. (B) Amplification score along Chromosome 8p for the 20 CIS cases. Regions of amplification were defined for each case and summarized across the group to determine the incidence of occurrence. Dashed lines represent the positions of *BRF2*, *WHSC1LC*, and *FGFR1* from top to bottom respectively. (C) Array CGH copy number profiles for two individual CIS cases with 8p amplification. Each black dot represents an array element ordered by genomic position. Those shifted to the left of the middle line (N) have decreased copy number (Del), whereas those shifted to the right have increased copy number (Amp). Dashed lines represent the positions of the three genes as in (B). The region highlighted in orange represents the region of high-level amplification in each sample. The amplicon in CIS1 includes only *BRF2* with *WHSC1L1* and *FGFR1* outside or spanning the boundaries while the amplicon in CIS2 contains all three genes. (D) Immunostaining of CIS2 with anti-*BRF2* polyclonal antibody revealed elevated staining in CIS epithelia compared with normal from the same tissue section.

**Figure 5:**
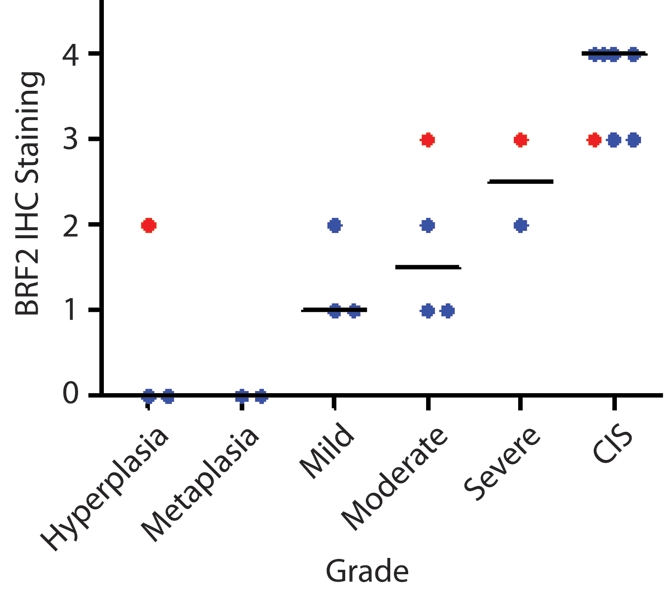
BRF2 expression in SqCC precancerous stages. Immunostaining of 21 lung SqCC precursor lesions with anti-*BRF2* polyclonal antibody revealed a monotonic increase in BRF2 expression with increasing histopathology grade. The area within the diagnostic area was scored as follows: 0, no positive staining; 1<25% positive cells; 2≤50% positive cells; 3≤75% positive cells; and 4<100% positive cells. Each sample is represented by a single dot above its corresponding grade with the horizontal black lines representing the median IHC score for each grade. Red samples highlight multiple grades taken from the same individual patient.

The cell type restricted pattern of activation coupled with its transformation potential strongly implicates *BRF2* as a lineage-specific oncogene in lung SqCC. SqCC carcinogenesis is thought to be a multistep process that involves the transformation of normal mucosa though a continuous range of precursor lesions up to CIS before invasive cancer and finally metastasis [52]. However, since most studies focus on clinically evident tumors, little is known about the molecular events preceding the development of lung cancer and the underlying basis of carcinogenesis. Unlike low grade dysplastic lung lesions that rarely progress, the majority of CIS cases will become invasive cancer [52]. Therefore, we hypothesized that critical alterations necessary for disease progression would be evident in preinvasive CIS lesions and persist in invasive tumors. To determine if *BRF2* activation occurs early in SqCC development (hypothesis 5), we analyzed gene dosage in a panel of 20 CIS lesions (Table S1, sample set 5) obtained by autofluorescence bronchoscopy (Methods). Remarkably, array CGH revealed *BRF2* copy number increases in the majority of CIS cases (Figure 4a) with 35% (seven out of 20) demonstrating high-level amplification (log_2_ ratio >0.8; Figure 4b). *WHSC1L1* and *FGFR1* were only amplified five times (five out of 20) and once (one out of 20), respectively, further excluding these genes as primary driver genes of the amplicon (Figure 4b and 4c). To confirm that amplification results in increased expression of *BRF2* in preinvasive lesions, we performed immunohistochemistry (IHC) on a CIS sample (CIS2) with amplification (Figure 4c). As expected, BRF2 expression was elevated in CIS epithelia in this sample in comparison to normal epithelia from the same patient (Figure 4d). Strong BRF2 expression was also observed in additional CIS cases with lower levels in earlier stages of neoplastic progression (mild, moderate, and severe dysplasia) and little or no staining in benign lesions (hyperplasia and metaplasia), confirming that gene activation is an early event in SqCC development (Figure 5). Interestingly, the only benign lesion in which BRF2 expression was observed was obtained from a patient that had also developed CIS (Figure 5). The high frequency of activation in preinvasive lesions suggests that BRF2 plays a critical role in the development of SqCC through the increase of cell growth potential. Since patient survival can be significantly improved if the lesions are detected and treated at their preinvasive stage, the identification of genes involved in the development of CIS and invasive SqCC is of vital clinical importance [52],[53]. Our finding that *BRF2* is a lineage-specific oncogene amplified early in SqCC development, and not expressed in normal lung tissue, represents a critical step in understanding the development of SqCC, and represents a promising target for therapeutic intervention.

### Increased RNA Processing Is Associated with BRF2 Overexpression

To identify other genes and functions that may be associated with *BRF2*-mediated initiation of tumorigenesis, we performed significance analysis of microarrays (SAM) on a panel of 111 NSCLC tumors (Table S1, sample set 2), followed by gene enrichment analysis using ingenuity pathway assist (IPA) (Methods). This analysis revealed 86 genes, which were significantly increased (78) or decreased (8) (false discovery rate <5%) in tumors with the highest *BRF2* expression (Table S6). IPA analysis revealed enrichment for genes with diverse biological functions including RNA post-transcriptional modification, gene expression, cell cycle, and cancer (Table S7). The identification of RNA post-transcriptional modification as the most significantly affected function (*p* = 1.7×10^−06^−4.73×10^−02^, the two significance values refer to a range of specific subfunctions) was significant, as this is one of the main roles of Pol III-related transcripts as stated above. The genes related to this function, which are increased in expression, include *FBL*, *CPSF6*, *RRP9*, *SNRPA*, *SFRS10*, *CSTF2T*, *LSM1*, and *CPSF3*, and are involved in the modification, polyadenylation, and processing of both mRNA and rRNA. Since these are fundamental processes necessary for proper protein production and therefore cell growth, upregulation of these components may be associated with the increased proliferative capacity of SqCC cells upon *BRF2* activation. However, the exact nature of this association is currently unknown and future studies will be needed to understand the mechanism responsible for BRF2-induced cell growth in SqCC.

Interestingly, as shown above, BRF2 activation leads to increased transcription from type 3 Pol III promoters that are involved in the transcription of snRNA genes including U6 and 7SK [49],[50]. snRNAs are responsible for a range of regulatory functions, including the alteration of gene expression and a potential role for snRNAs in the genomic instability of cancer that has been proposed [54]. In particular, U6 snRNA forms the catalytic core of the spliceosome [55]. The spliceosome performs the splicing of precursor mRNA in eukaryotic cells, removing introns and joining exons. This process is tightly regulated during growth and development and aberrant splicing has been linked to numerous human diseases, including cancer [56]. In fact, many oncogenes demonstrate alternative splicing patterns associated with neoplasia, and splicing regulatory factor expression levels have been shown to increase during cancer progression. Strikingly, many of the genes we identified as being associated with increased *BRF2* expression, including *SNRPA* and *SFRS10*, are known to interact with snRNAs including U6 in the spliceosome complex. In addition, *SNAPC5*, which encodes a member of the snRNA-activating complex that is required in conjunction with BRF2 to initiate transcription from snRNA promoters [57], was also found to be increased in samples with high BRF2 expression. Taken together, our data suggest that BRF2-mediated increase of U6 as well as other splicing regulatory factors may contribute to oncogenesis in SqCC with 8p amplification. Future studies of the role BRF2 overexpression plays in spliceosome function will yield insight into this potential function, and its role in the neoplastic transformation of lung epithelium to SqCC.

### Association of BRF2 with Clinical-Pathological and Genomic Features

Lastly, to investigate the potential clinical significance of BRF2 activation in patients with lung SqCC and better characterize this subgroup of tumors, we next sought to determine the association between 8p amplification and clinical-pathological and genetic features. For this purpose, we expanded our sample set to include 92 SqCC tumors with well-annotated clinical information that were analyzed by tiling-path array CGH. Overall, increase of *BRF2* copy number was found in 43% of the expanded dataset, in concordance with the original frequency of alteration. No associations were found between age, gender, smoking status, or stage and *BRF2* amplification in our dataset. Furthermore, no significant associations between the level of *BRF2* expression and patient survival were seen in two independent datasets (Figure S6). However, SqCC tumors with and without *BRF2* activation showed a unique genome-wide spectrum of DNA amplifications, suggesting that different genetic pathways may be involved in their development (Table S8). Assessment of other clinical and genetic features—for example response to therapy and mutation events—will be necessary in the future to further explore the characteristics of SqCC patients harboring BRF2 amplification.

## Discussion

In summary, we show here that the focal amplification of Chromosome 8p12, one of the most frequent amplification events in NSCLC, plays a key role in squamous cell lineage specificity of the disease. Through the integration of genetic and gene expression data for >330 clinical tumor specimens in conjunction with functional cell model studies, we identified *BRF2* as the target of this amplification and a cell lineage-specific oncogene, the only such oncogene described for lung SqCC to date. In addition, we highlight the oncogenic potential of *BRF2* for the first time and associate its activation with increased Pol III activity, RNA processing, and resultant cell growth potential.

The lineage-dependence model suggests that cancer cells rely on the constitutive activation of lineage-regulating genes involved in normal development for their continued survival and proliferation [7]. *BRF2* is unique in that it is not a prototypical lineage-specific oncogene as no role in normal lineage development has been established. These data suggest that lineage-specific oncogenes may span numerous biological functions, and they are not limited only to the established class of transcription factors (lineage survival oncogenes) discovered to date, but also a class of genes selected in tumorigenesis in a cell lineage-specific manner. Recently, a candidate a lineage survival oncogene for lung AC, *TITF1* (thyroid transcription factor 1), has also been described by numerous groups [16],[58]–[60]. However, high-level amplification of *TITF1* and concordant increase in protein levels occur at approximately the same frequency in both AC and SqCC (∼10%–15%, although protein levels are higher in AC overall) [61],[62]. Thus, BRF2 seems to be even more specific in terms of genetic alteration and its association with an individual subtype. Likewise, *SOX2* has been identified as a lineage survival oncogene in lung and esophageal SqCCs [63]. Of interest, both SqCC with and without *BRF2* amplification also contains amplification of Chromosome arm 3q targeting *SOX2*, suggesting that tumors with *BRF2* amplification represent a unique subset within the larger SqCC group (Table S8). Nonetheless, our results combined with the recent discoveries of *TITF1* and *SOX2* suggest that the genes required to initiate tumorigenesis in distinct biological contexts may shape the preferential selection of amplifications and resulting phenotypes specific to different cancers, highlighting the opportunity for treatment design targeting specific cell type.

## Supporting Information

Figure S1 **qRT-PCR analysis of** ***BRF2*** **expression levels in SqCC tumors with and without gene dosage increases.** *BRF2* expression levels were determined for 16 SqCC tumors with matching array CGH data using TaqMan analysis as described in the Methods section. Normalized *BRF2* expression values were compared between each sample and a normal lung reference to determine the relative fold change. Raw data from these experiments are provided in Table S4. (a) The expression for each individual tumor is plotted along with its corresponding case number. Samples are organized according to their *BRF2* copy number status as determined by array CGH (see Methods). The white bars represent tumors without copy number increase, whereas the crosshatched bars represent those with copy number increase. SqCC tumors with copy number gain/amplification have higher expression than tumors without. (b) Box plots representing the expression of *BRF2* in SqCC tumors with and without gain/amplification. The average *BRF2* expression is significantly higher in SqCC tumors with amplification/gain than in those without (*p* = 0.0003, one-tailed Mann-Whitney U test), confirming the findings from the microarray experiments detailed in the text.
(0.33 MB PDF)

Figure S2 **qRT-PCR analysis of** ***BRF2*** **expression levels in NSCLC cell lines.** *BRF2* expression levels were determined for 20 NSCLC cell lines with matching array CGH data using TaqMan analysis as described in the Methods section. Normalized *BRF2* expression values were compared between each sample and a normal lung reference to determine the relative fold change. Raw data from these experiments are provided in Table S5. (A) *BRF2* expression across NSCLC cell lines. Samples are organized according to their histological subtype. The white bars represent AC cell lines, whereas the crosshatched bars represent SqCC cell lines. The two SqCC samples with high-level *BRF2* amplification (HCC95 and H520) also have the highest *BRF2* transcript levels. (B) *BRF2* expression is strongly correlated with gene dosage. Log_2_ array CGH ratios for *BRF2* are plotted on the *y*-axis with the corresponding *BRF2* mRNA expression (as determined by qRT-PCR, described above) for each cell line plotted on the *x*-axis (Pearson *r* = 0.8645, *p*<0.0001).
(0.35 MB PDF)

Figure S3 ***BRF2*** **knockdown reduces U6 levels in H520 cells.** (A) U6 snRNA and 5S rRNA levels were determined by Northern blot for H520 cells expressing either *BRF2* targeting shRNA (BRF2-1) or a vector control (see Methods). (B) Gel images were analyzed with *ImageJ* software and the background corrected pixel densities for U6 were normalized to 5S for each sample. The resulting ratios are plotted relative to the control, demonstrating ∼35% decrease in U6 levels in the cell line expressing the *BRF2* shRNA.
(0.31 MB PDF)

Figure S4 **The effect of** ***BRF2*** **knockdown is specific to SqCC cell lines with amplification.** (A) *BRF2* copy number (array CGH) and (B) expression (qRT-PCR) in H520, HCC2450, and HCC15 lung SqCC cell lines. (C) Decrease in *BRF2* mRNA levels in H520, HCC2450, and HCC15 cells expressing shRNA targeting *BRF2* (*BRF2-1*) relative to those expressing respective negative vector controls (mean ± SEM of duplicate experiments). (D) *BRF2* knockdown results in significantly decreased cell proliferation in H520 compared to HCC2450 and HCC15 as measured by MTT assay relative to respective vector controls (mean ± SEM of duplicate experiments). Values plotted are from day 5 measurements (Methods). * *p*<0.05 (ANOVA).
(0.36 MB PDF)

Figure S5 **qRT-PCR analysis of** ***BRF2*** **expression levels in stably transduced immortalized HBEC lines.** *BRF2* expression levels were determined using TaqMan analysis as described in the Methods section. Normalized *BRF2* expression values were compared between each cell line and their respective vector controls and the corresponding fold change is plotted.
(0.26 MB PDF)

Figure S6 **Association of** ***BRF2*** **expression and patient survival in lung SqCC.** Publically available datasets from the Gene Expression Omnibus, (A) GSE3141 and (B) GSE4573, were used to assess the association of *BRF2* expression and patient survival. The survival distributions of the top 40% and bottom 40% of samples, on the basis of expression of *BRF2*, were compared using a Kaplan-Meier analysis. These cutoffs (top and bottom 40%) were picked to reflect the frequency of *BRF2* copy number increase in lung SqCC (40% of all cases). *p*-Values for comparing survival distributions were calculated using the Mantel-Cox method (log-rank test). Kaplan Meier analysis was performed using the Mathworks MATLAB Statistics toolbox. Mantel-Cox p-values were calculated in MATLAB using the following file: http://www.mathworks.com/matlabcentral/fileexchange/22317-logrank.
(18.34 MB PDF)

Table S1 **Clinical samples used in analyses.**
(0.04 MB DOC)

Table S2 **Regions of copy number difference on Chromosome arm 8p between AC and SqCC.**
(0.03 MB DOC)

Table S3 **Genes differentially expressed between AC and SqCC with regions of copy number difference.**
(0.04 MB DOC)

Table S4 **Raw qRT-PCR data for SqCC tumor samples.**
(0.04 MB DOC)

Table S5 **Raw qRT-PCR data for NSCLC cell lines.**
(0.12 MB DOC)

Table S6 **BRF2 expression signature.**
(0.05 MB DOC)

Table S7 **Cellular functions enriched in NSCLC tumors with high** ***BRF2*** **expression.**
(0.08 MB DOC)

Table S8 **High-level amplifications associated with SqCC tumors with and without BRF2 activation.**
(0.04 MB DOC)

## Abbreviations

AC: adenocarcinoma
CGH: comparative genomic hybridization
CIS: carcinoma in situ
HBEC: human bronchial epithelial cell
NSCLC: non-small cell lung cancer
qRT-PCR: quantitative reverse-transcriptase PCR
RNAi: RNA interference
SEM: standard error measure
shRNA: short hairpin RNA
siRNA: small interfering RNA
snRNA: small nuclear RNA
SqCC: squamous cell carcinoma
