# Supplementary figures and images for: Integrative Genomic Analyses Identify BRF2 as a Novel Lineage-Specific Oncogene in Lung Squamous Cell Carcinoma
Source: PLoS Med. 2010 Jul 27;7(7):e1000315. doi: 10.1371/journal.pmed.1000315 (PMC2910599; doi:10.1371/journal.pmed.1000315)

Figure S1

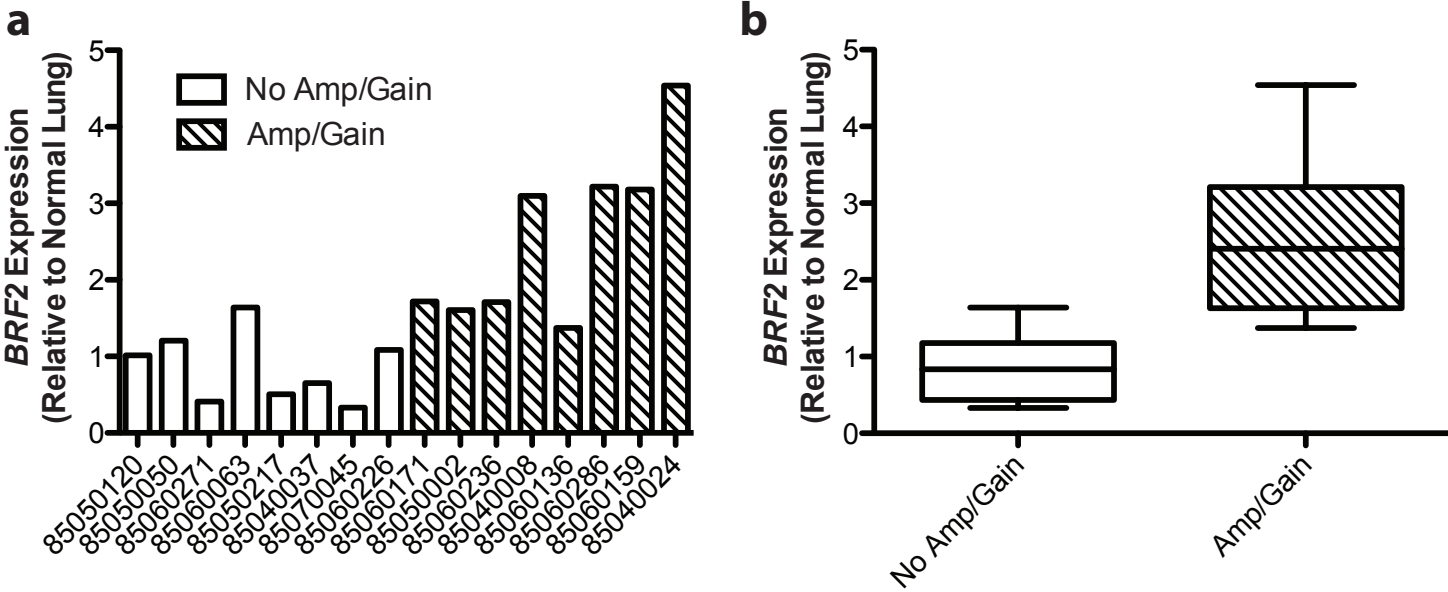

Supplement: Figure S1 — qRT-PCR analysis of BRF2 expression levels in SqCC tumors with and without gene dosage increases. BRF2 expression levels were determined for 16 SqCC tumors with matching array CGH data using TaqMan analysis as described in the Methods section. Normalized BRF2 expression values were compared between each sample and a normal lung reference to determine the relative fold change. Raw data from these experiments are provided in Table S4. (a) The expression for each individual tumor is plotted along with its corresponding case number. Samples are organized according to their BRF2 copy number status as determined by array CGH (see Methods). The white bars represent tumors without copy number increase, whereas the crosshatched bars represent those with copy number increase. SqCC tumors with copy number gain/amplification have higher expression than tumors without. (b) Box plots representing the expression of BRF2 in SqCC tumors with and without gain/amplification. The average BRF2 expression is significantly higher in SqCC tumors with amplification/gain than in those without (p = 0.0003, one-tailed Mann-Whitney U test), confirming the findings from the microarray experiments detailed in the text. (0.33 MB PDF) [file pmed.1000315.s001.pdf]

Figure S2

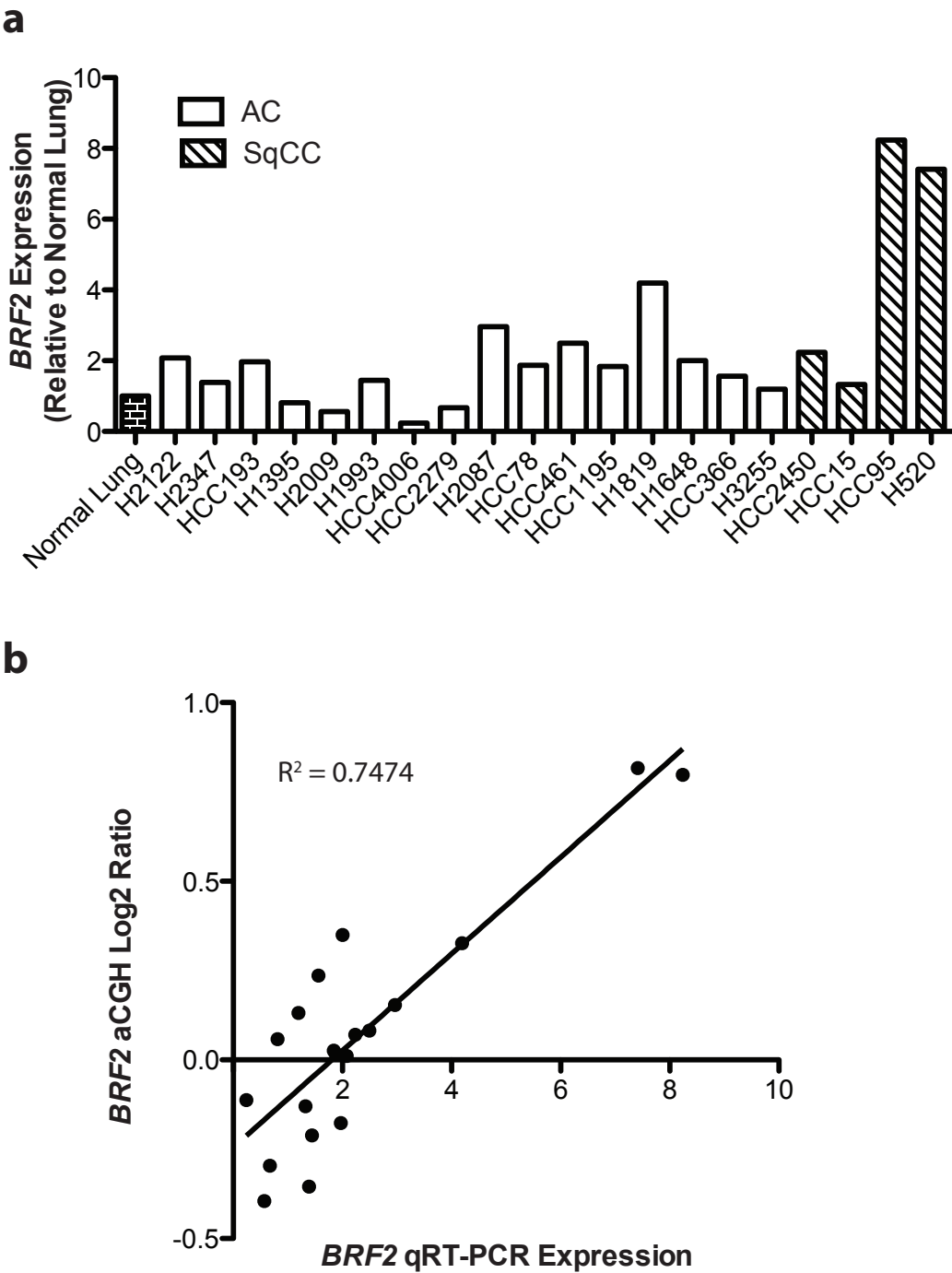

Supplement: Figure S2 — qRT-PCR analysis of BRF2 expression levels in NSCLC cell lines. BRF2 expression levels were determined for 20 NSCLC cell lines with matching array CGH data using TaqMan analysis as described in the Methods section. Normalized BRF2 expression values were compared between each sample and a normal lung reference to determine the relative fold change. Raw data from these experiments are provided in Table S5. (A) BRF2 expression across NSCLC cell lines. Samples are organized according to their histological subtype. The white bars represent AC cell lines, whereas the crosshatched bars represent SqCC cell lines. The two SqCC samples with high-level BRF2 amplification (HCC95 and H520) also have the highest BRF2 transcript levels. (B) BRF2 expression is strongly correlated with gene dosage. Log2 array CGH ratios for BRF2 are plotted on the y-axis with the corresponding BRF2 mRNA expression (as determined by qRT-PCR, described above) for each cell line plotted on the x-axis (Pearson r = 0.8645, p<0.0001). (0.35 MB PDF) [file pmed.1000315.s002.pdf]

**Figure S3**

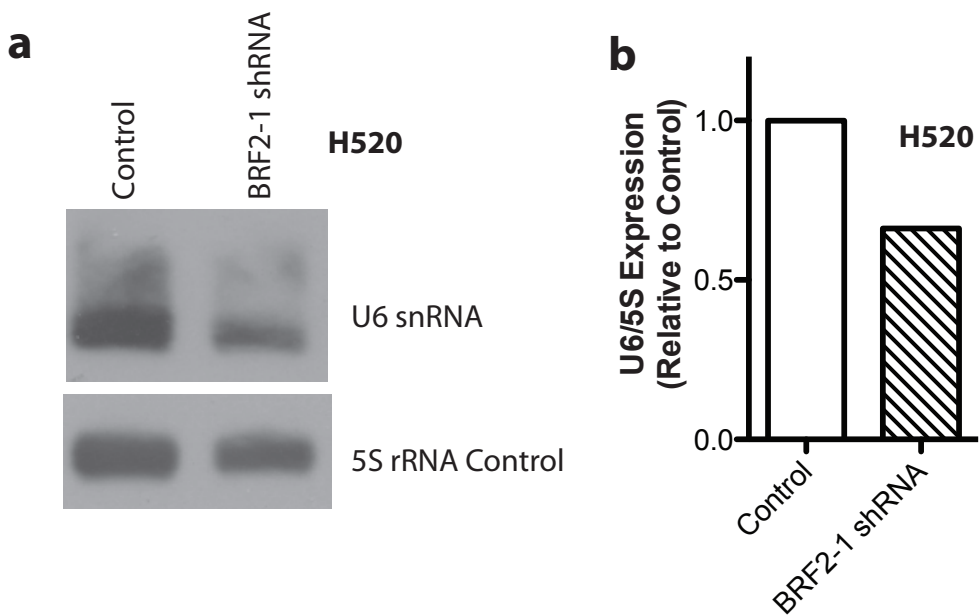

Supplement: Figure S3 — BRF2 knockdown reduces U6 levels in H520 cells. (A) U6 snRNA and 5S rRNA levels were determined by Northern blot for H520 cells expressing either BRF2 targeting shRNA (BRF2-1) or a vector control (see Methods). (B) Gel images were analyzed with ImageJ software and the background corrected pixel densities for U6 were normalized to 5S for each sample. The resulting ratios are plotted relative to the control, demonstrating ∼35% decrease in U6 levels in the cell line expressing the BRF2 shRNA. (0.31 MB PDF) [file pmed.1000315.s003.pdf]

**Figure S4**

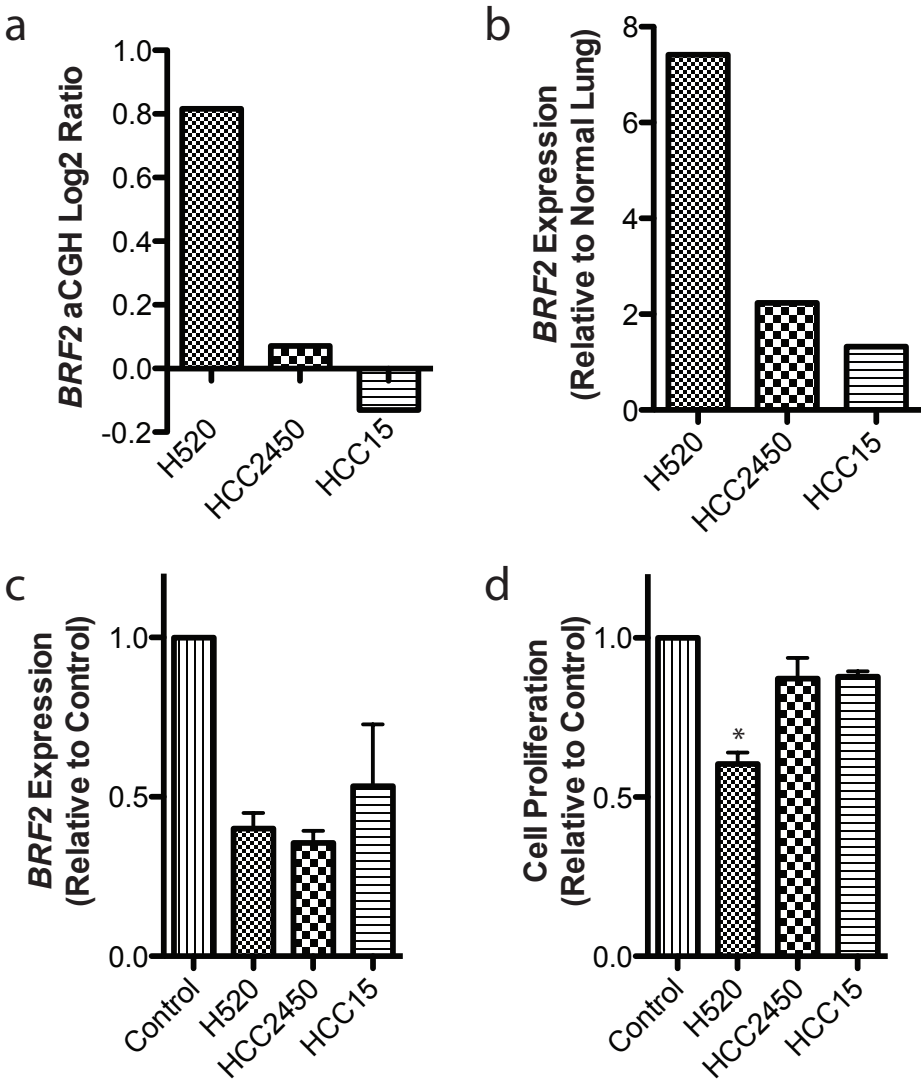

Supplement: Figure S4 — The effect of BRF2 knockdown is specific to SqCC cell lines with amplification. (A) BRF2 copy number (array CGH) and (B) expression (qRT-PCR) in H520, HCC2450, and HCC15 lung SqCC cell lines. (C) Decrease in BRF2 mRNA levels in H520, HCC2450, and HCC15 cells expressing shRNA targeting BRF2 (BRF2-1) relative to those expressing respective negative vector controls (mean ± SEM of duplicate experiments). (D) BRF2 knockdown results in significantly decreased cell proliferation in H520 compared to HCC2450 and HCC15 as measured by MTT assay relative to respective vector controls (mean ± SEM of duplicate experiments). Values plotted are from day 5 measurements (Methods). * p<0.05 (ANOVA). (0.36 MB PDF) [file pmed.1000315.s004.pdf]

**Figure S5**

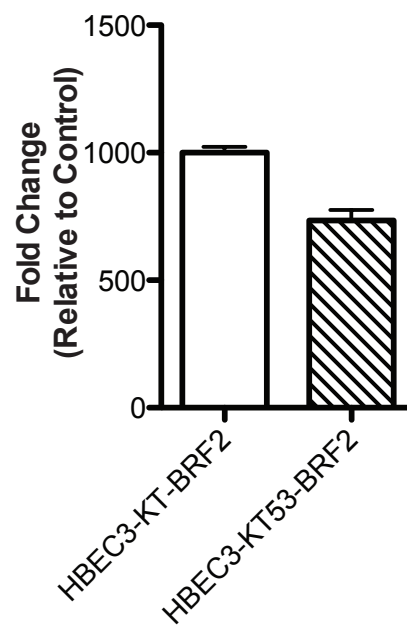

Supplement: Figure S5 — qRT-PCR analysis of BRF2 expression levels in stably transduced immortalized HBEC lines. BRF2 expression levels were determined using TaqMan analysis as described in the Methods section. Normalized BRF2 expression values were compared between each cell line and their respective vector controls and the corresponding fold change is plotted. (0.26 MB PDF) [file pmed.1000315.s005.pdf]

Figure S6

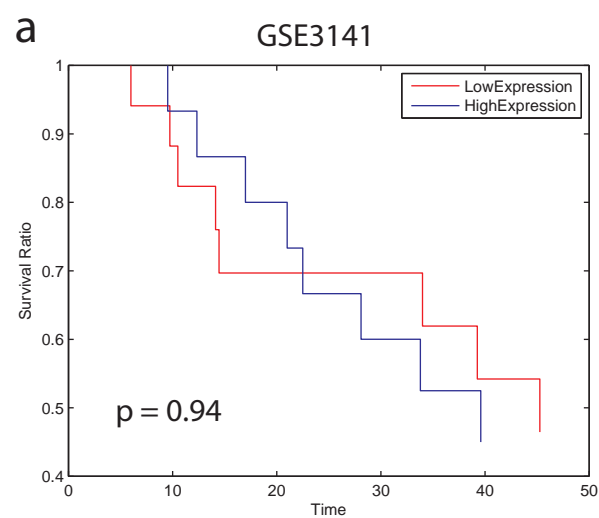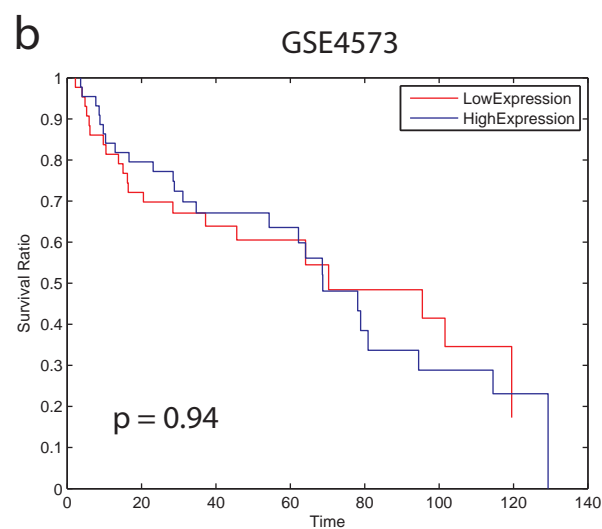

Supplement: Figure S6 — Association of BRF2 expression and patient survival in lung SqCC. Publically available datasets from the Gene Expression Omnibus, (A) GSE3141 and (B) GSE4573, were used to assess the association of BRF2 expression and patient survival. The survival distributions of the top 40% and bottom 40% of samples, on the basis of expression of BRF2, were compared using a Kaplan-Meier analysis. These cutoffs (top and bottom 40%) were picked to reflect the frequency of BRF2 copy number increase in lung SqCC (40% of all cases). p-Values for comparing survival distributions were calculated using the Mantel-Cox method (log-rank test). Kaplan Meier analysis was performed using the Mathworks MATLAB Statistics toolbox. Mantel-Cox p-values were calculated in MATLAB using the following file: http://www.mathworks.com/matlabcentral/fileexchange/22317-logrank. (18.34 MB PDF) [file pmed.1000315.s006.pdf]
