# Supplementary material for: Integrative Genomic Analyses Identify BRF2 as a Novel Lineage-Specific Oncogene in Lung Squamous Cell Carcinoma
Source: PLoS Med. 2010 Jul 27;7(7):e1000315. doi: 10.1371/journal.pmed.1000315 (PMC2910599; doi:10.1371/journal.pmed.1000315)
Supplement: Table S1 — Clinical samples used in analyses. (0.04 MB DOC) [file pmed.1000315.s007.doc]

**Table S1:** Clinical Samples used in Analyses

| **Sample Set** | **Cohort** | **# of Samples** | **# AC** | **# SqCC** | **# Other NSCLC** | **# Non-Neoplasitic Lung Tissue**^$^ | **Carcinoma in Situ** | **Assay Type** |
| --- | --- | --- | --- | --- | --- | --- | --- | --- |
| 1a | BC Cancer Agency - Vancouver  St. Paul's Hospital - Vancouver  University Health Network - Toronto | 161 | 103 | 58 | 0 | 0 | 0 | Copy Number - BCCRC Whole Genome Tiling Path Array CGH |
| 1b* | BC Cancer Agency - Vancouver | 47 | 34 | 13 | 0 | 0 | 0 | Gene Expression - Custom Agilent Whole Genome |
| 2 | GEO Duke University – GSE3141 | 111 | 58 | 53 | 0 | 0 | 0 | Gene Expression - Affymetrix GeneChip Human Genome U133 Plus 2.0 Array |
| 3 | BC Cancer Agency – Vancouver | 0 | 0 | 0 | 0 | 67 | 0 | Gene Expression - Affymetrix GeneChip Human Genome U133 Plus 2.0 Array |
| 4 | University Health Network - Toronto | 157 | 77 | 33 | 8 | 39 | 0 | Gene Expression -qPCR |
| 5 | BC Cancer Agency - Vancouver | 20 | 0 | 0 | 0 | 0 | 20 | Copy Number - BCCRC Whole Genome Tiling Path Array CGH |

*Sample set 1b is a subset of 1a that was also analyzed for expression

^$^ Non-neoplastic lung tissue includes exfoliated bronchial epithelial cells from cancer free individuals
