# Supplementary material for: Integrative Genomic Analyses Identify BRF2 as a Novel Lineage-Specific Oncogene in Lung Squamous Cell Carcinoma
Source: PLoS Med. 2010 Jul 27;7(7):e1000315. doi: 10.1371/journal.pmed.1000315 (PMC2910599; doi:10.1371/journal.pmed.1000315)
Supplement: Table S2 — Regions of copy number difference on Chromosome arm 8p between AC and SqCC. (0.03 MB DOC) [file pmed.1000315.s008.doc]

**Table S2:** Regions of Copy Number Difference on Chromosome Arm 8p between AC and SqCC

| Region | Base Pair Start* | Base Pair End* | Size (Mbp) | Predominant Status | Genes |
| --- | --- | --- | --- | --- | --- |
| 1 | 36402463 | 41740524 | 5.34 | Gained SqCC | *KCNU1, FKSG2, ZNF703, SPFH2, PROSC, GPR124, BRF2, RAB11FIP1, MGC33309, ADRB3, EIF4EBP1, ASH2L, STAR, LSM1, BAG4, DDHD2, HTPAP, WHSC1L1, LETM2, FGFR1, FLJ43582, TACC1, HTRA4, TM2D2, ADAM9, ADAM32, ADAM18, ADAM2, INDO, C8orf4, ZMAT4, SFRP1, GOLGA7, SLD5, DKFZp586M1819, FLJ25169, ANK1* |
| 2 | 42132418 | 42442770 | 0.31 | Gained SqCC | *AP3M2, PLAT, IKBKB, POLB, DKK4, VDAC3, SLC20A2* |

* Hg18 (March 2006) build genomic coordinates
