# Supplementary material for: Integrative Genomic Analyses Identify BRF2 as a Novel Lineage-Specific Oncogene in Lung Squamous Cell Carcinoma
Source: PLoS Med. 2010 Jul 27;7(7):e1000315. doi: 10.1371/journal.pmed.1000315 (PMC2910599; doi:10.1371/journal.pmed.1000315)
Supplement: Table S3 — Genes differentially expressed between AC and SqCC with regions of copy number difference. (0.04 MB DOC) [file pmed.1000315.s009.doc]

**Table S3:** Genes Differentially Expressed between AC and SqCC with Regions of Copy Number Difference

| **Gene Symbol** | **Base Pair Start*** | **Base Pair End*** | **AC vs SqCC *p*-Value** | **Spearman Correlation** | **Spearman *p*-Value** | **SqCC Amp vs SqCC Neutral *p*-Value** | **SqCC Tumors vs Normal Bronchial Cells *p*-Value** | **SqCC Tumors vs Normal Bronchial Cells Fold Change** |
| --- | --- | --- | --- | --- | --- | --- | --- | --- |
| *AP3M2* | 42129760 | 42147858 | 0.004778371 | 0.68681 | 0.008585714 | 0.090916667 | 0 | 0.619113864 |
| *ASH2L* | 38082222 | 38116216 | 0.006827223 | 0.81319 | 0.002066667 | 0.0476 | 0.002228668 | 1.414206566 |
| *BRF2* | 37820560 | 37826569 | 0.005604416 | 0.87363 | 0.00015 | 0.0476 | 0 | 2.065624233 |
| *GOLGA7* | 41467237 | 41487656 | 0.007275417 | 0.73626 | 0.004816667 | 0.037875 | 0.016866738 | 1.253440175 |
| *LSM1* | 38140014 | 38153183 | 0.004997841 | 0.8956 | 0 | 0.0476 | 0.015574938 | 1.447210583 |
| *POLB* | 42315186 | 42348470 | 0.013201801 | 0.36264 | 0.11199 | 0.090916667 | 0.01596931 | 1.09616831 |
| *SLC20A2* | 42393149 | 42516225 | 0.010014008 | 0.56593 | 0.0295875 | 0.37753 | 0.00003691 | 0.741931537 |
| *TM2D2* | 38965483 | 38973198 | 0.000005642 | 0.81319 | 0.002066667 | 0.0784625 | 0.120328001 | 1.398021442 |
| *WHSC1L1* | 38251717 | 38358947 | 0.012820856 | 0.75103 | 0.00308 | 0.05714 | 0.134275652 | 1.265142199 |
| *ZNF703* | 37672458 | 37675554 | 0.000276055 | 0.43956 | 0.075011111 | 0.095233333 | 0.437252534 | 1.342126264 |

Note: All p-values are corrected for multiple comparisons

* * Hg18 (March 2006) build genomic coordinates
