# Supplementary material for: Integrative Genomic Analyses Identify BRF2 as a Novel Lineage-Specific Oncogene in Lung Squamous Cell Carcinoma
Source: PLoS Med. 2010 Jul 27;7(7):e1000315. doi: 10.1371/journal.pmed.1000315 (PMC2910599; doi:10.1371/journal.pmed.1000315)
Supplement: Table S4 — Raw qRT-PCR data for SqCC tumor samples. (0.04 MB DOC) [file pmed.1000315.s010.doc]

**Table S4:** Raw qRT-PCR Data for SqCC Tumor Samples

| **Sample** | **Subtype** | **BRF2 Amplification/Gain** | **Average Threshold Cycle (Ct) BRF2 (triplicates)** | **(Average Ct of 18S) – BRF2** |
| --- | --- | --- | --- | --- |
| 85050120 | SqCC | No | 30.21713011 | 20.89273373 |
| 85060171 | SqCC | Yes | 28.20661699 | 20.13349471 |
| 85050217 | SqCC | No | 31.1414772 | 21.89197045 |
| 85040037 | SqCC | No | 30.39563626 | 21.528836 |
| 85050002 | SqCC | Yes | 28.83488594 | 20.23183328 |
| 85060236 | SqCC | Yes | 28.59149168 | 20.1397765 |
| 85070045 | SqCC | No | 30.9453302 | 22.50096622 |
| 85040008 | SqCC | Yes | 27.95393517 | 19.2841258 |
| 85050050 | SqCC | No | 29.01378589 | 20.64421962 |
| 85060136 | SqCC | Yes | 29.35566569 | 20.45836176 |
| 85060286 | SqCC | Yes | 28.57203476 | 19.22790502 |
| 85060226 | SqCC | No | 29.1753359 | 20.7968551 |
| 85060159 | SqCC | Yes | 27.83335017 | 19.2463581 |
| 85040024 | SqCC | Yes | 27.53002564 | 18.73251462 |
| 85060271 | SqCC | No | 31.00386335 | 22.20660446 |
| 85060063 | SqCC | No | 29.34551376 | 20.20263252 |
