# Supplementary material for: Integrative Genomic Analyses Identify BRF2 as a Novel Lineage-Specific Oncogene in Lung Squamous Cell Carcinoma
Source: PLoS Med. 2010 Jul 27;7(7):e1000315. doi: 10.1371/journal.pmed.1000315 (PMC2910599; doi:10.1371/journal.pmed.1000315)
Supplement: Table S5 — Raw qRT-PCR data for NSCLC cell lines. (0.12 MB DOC) [file pmed.1000315.s011.doc]

**Table S6:** BRF2 Expression Signature

| **Gene Symbol** | **Gene Name** | **Fold Change** | **q-value (%)** |
| --- | --- | --- | --- |
| **Down Regulated Genes** | | | |
| *OLR1* | Oxidised low density lipoprotein (lectin-like) receptor 1 | 0.452060785 | 2.277015 |
| *PAG1* | phosphoprotein associated with glycosphingolipid microdomains 1 | 0.235313993 | 2.277015 |
| *ACO1* | "aconitase 1, soluble" | 0.508458874 | 4.626315 |
| *ALDH3B1* | "aldehyde dehydrogenase 3 family, member B1" | 0.458373487 | 4.626315 |
| *BTD* | biotinidase | 0.462978889 | 4.626315 |
| *EPOR* | erythropoietin receptor | 0.633429027 | 4.626315 |
| *HYAL2* | hyaluronoglucosaminidase 2 | 0.575233608 | 4.626315 |
| *SH3KBP1* | SH3-domain kinase binding protein 1 | 0.44140154 | 4.626315 |
| **Up Regulated Genes** | | | |
| *ALDOC* | "aldolase C, fructose-bisphosphate" | 3.021807831 | 0 |
| *BAG4* | BCL2-associated athanogene 4 | 4.512803347 | 0 |
| *BRF2* | "BRF2, subunit of RNA polymerase III transcription initiation factor, BRF1-like" | 4.258993609 | 0 |
| *CPSF3* | "cleavage and polyadenylation specific factor 3, 73kDa" | 1.706311305 | 0 |
| *E2F6* | E2F transcription factor 6 | 1.943479519 | 0 |
| *EMD* | emerin (Emery-Dreifuss muscular dystrophy) | 1.796811177 | 0 |
| *GMEB2* | glucocorticoid modulatory element binding protein 2 | 1.530363907 | 0 |
| *GTF2F2* | "general transcription factor IIF, polypeptide 2, 30kDa" | 1.95124788 | 0 |
| *LOC388796* | "RNA, U71A small nucleolar /// Hypothetical LOC388796" | 1.606194625 | 0 |
| *LSM1* | "LSM1 homolog, U6 small nuclear RNA associated (S. cerevisiae)" | 3.189739084 | 0 |
| *MLF2* | myeloid leukemia factor 2 | 1.779729184 | 0 |
| *PRPF19* | PRP19/PSO4 pre-mRNA processing factor 19 homolog (S. cerevisiae) | 2.006609962 | 0 |
| *PSARL* | "presenilin associated, rhomboid-like" | 2.002541253 | 0 |
| *SPATA19* | spermatogenesis associated 19 | 2.489527522 | 0 |
| *ASH2L* | "ash2 (absent, small, or homeotic)-like (Drosophila)" | 2.84748201 | 3.312021 |
| *DNAJC14* | "DnaJ (Hsp40) homolog, subfamily C, member 14" | 1.517869115 | 3.312021 |
| *EIF4EBP1* | eukaryotic translation initiation factor 4E binding protein 1 | 4.682529049 | 3.312021 |
| *EYA2* | eyes absent homolog 2 (Drosophila) | 4.670844469 | 3.312021 |
| *FBL* | fibrillarin /// fibrillarin | 2.134799698 | 3.312021 |
| *FLJ40869* | hypothetical protein FLJ40869 | 2.432050237 | 3.312021 |
| *FLJ46072* | FLJ46072 protein | 2.822480777 | 3.312021 |
| *MGC14798* | similar to RIKEN cDNA 5730421E18 gene | 2.38168193 | 3.312021 |
| *MRPS18A* | mitochondrial ribosomal protein S18A | 1.615538192 | 3.312021 |
| *PIK3CB* | "phosphoinositide-3-kinase, catalytic, beta polypeptide" | 1.684545021 | 3.312021 |
| *POLS* | polymerase (DNA directed) sigma | 1.683268347 | 3.312021 |
| *PPAPDC1B* | Phosphatidic acid phosphatase type 2 domain containing 1B | 3.952117941 | 3.312021 |
| *RNU3IP2* | "RNA, U3 small nucleolar interacting protein 2" | 2.737462956 | 3.312021 |
| *SHOX2* | short stature homeobox 2 | 2.903692145 | 3.312021 |
| *SLC6A15* | "solute carrier family 6, member 15" | 2.564480003 | 3.312021 |
| *SNAPC5* | "Small nuclear RNA activating complex, polypeptide 5, 19kDa" | 1.740680112 | 3.312021 |
| *STAR* | steroidogenic acute regulator | 9.706530618 | 3.312021 |
| *STK35* | serine/threonine kinase 35 | 2.243139166 | 3.312021 |
| *ZNF326* | zinc finger protein 326 | 1.88144622 | 3.312021 |
| *CSTF2T* | "cleavage stimulation factor, 3' pre-RNA, subunit 2, 64kDa, tau variant" | 1.81189461 | 3.554364 |
| *DNPEP* | aspartyl aminopeptidase | 1.425669432 | 3.554364 |
| *EXOSC4* | exosome component 4 | 1.858675749 | 3.554364 |
| *FLJ10774* | N-acetyltransferase-like protein | 2.021839845 | 3.554364 |
| *GSK3B* | Glycogen synthase kinase 3 beta | 1.483673544 | 3.554364 |
| *PAK2* | p21 (CDKN1A)-activated kinase 2 | 1.798321454 | 3.554364 |
| *POLR2H* | polymerase (RNA) II (DNA directed) polypeptide H | 2.051661834 | 3.554364 |
| *PSMC2* | "proteasome (prosome, macropain) 26S subunit, ATPase, 2" | 1.964111708 | 3.554364 |
| *SFRS10* | "splicing factor, arginine/serine-rich 10 (transformer 2 homolog, Drosophila)" | 1.700301079 | 3.554364 |
| *SH3GL3* | SH3-domain GRB2-like 3 | 2.277929868 | 3.554364 |
| *SNRPA* | small nuclear ribonucleoprotein polypeptide A | 1.941167567 | 3.554364 |
| *TPI1* | triosephosphate isomerase 1 | 1.712197453 | 3.554364 |
| *ASCL2* | achaete-scute complex-like 2 (Drosophila) | 4.742774577 | 4.626315 |
| *C20orf55* | chromosome 20 open reading frame 55 | 1.952250964 | 4.626315 |
| *C20orf72* | chromosome 20 open reading frame 72 | 2.390952311 | 4.626315 |
| *CBLL1* | Cas-Br-M (murine) ecotropic retroviral transforming sequence-like 1 | 1.783072174 | 4.626315 |
| *CCT5* | "chaperonin containing TCP1, subunit 5 (epsilon)" | 2.254814982 | 4.626315 |
| *CPSF6* | "cleavage and polyadenylation specific factor 6, 68kDa" | 1.525885407 | 4.626315 |
| *DKFZP564I1171* | DKFZP564I1171 protein | 2.558722201 | 4.626315 |
| *ERAL1* | Era G-protein-like 1 (E. coli) | 1.519335858 | 4.626315 |
| *FARSLB* | "phenylalanine-tRNA synthetase-like, beta subunit" | 1.626841418 | 4.626315 |
| *FLJ13149* | hypothetical protein FLJ13149 | 1.779875126 | 4.626315 |
| *GFM1* | "G elongation factor, mitochondrial 1" | 1.778617535 | 4.626315 |
| *GYS1* | glycogen synthase 1 (muscle) | 1.873267104 | 4.626315 |
| *HMGCS1* | 3-hydroxy-3-methylglutaryl-Coenzyme A synthase 1 (soluble) | 2.542545514 | 4.626315 |
| *HSPBP1* | hsp70-interacting protein | 1.678676593 | 4.626315 |
| *IDH2* | "isocitrate dehydrogenase 2 (NADP+), mitochondrial" | 2.393297923 | 4.626315 |
| *KIAA0888* | KIAA0888 protein | 3.926356103 | 4.626315 |
| *MGC9850* | hypothetical protein MGC9850 | 1.69715395 | 4.626315 |
| *MID1* | midline 1 (Opitz/BBB syndrome) | 3.045149472 | 4.626315 |
| *NAT5* | "N-acetyltransferase 5 (ARD1 homolog, S. cerevisiae)" | 1.925939343 | 4.626315 |
| *NUDT15* | nudix (nucleoside diphosphate linked moiety X)-type motif 15 | 2.00369167 | 4.626315 |
| *PCTK1* | PCTAIRE protein kinase 1 | 1.637893164 | 4.626315 |
| *PMS2L5* | postmeiotic segregation increased 2-like 5 | 1.521108189 | 4.626315 |
| *PTTG1* | pituitary tumor-transforming 1 | 2.190433325 | 4.626315 |
| *RECQL4* | RecQ protein-like 4 | 3.276435851 | 4.626315 |
| *RP11-529I10.4* | deleted in a mouse model of primary ciliary dyskinesia | 1.480653912 | 4.626315 |
| *SDHA /// SDHAL2* | "succinate dehydrogenase complex, subunit A, flavoprotein (Fp) /// succinate dehydrogenase complex, subunit A, flavoprotein-like 2" | 1.433713684 | 4.626315 |
| *SLC7A1* | "solute carrier family 7 (cationic amino acid transporter, y+ system), member 1" | 2.723386492 | 4.626315 |
| *TUBB* | "tubulin, beta polypeptide /// tubulin, beta polypeptide" | 1.567472957 | 4.626315 |
| *UNG* | uracil-DNA glycosylase | 2.30873151 | 4.626315 |
| *WBSCR1* | Williams-Beuren syndrome chromosome region 1 | 1.850560394 | 4.626315 |
| *YWHAQ* | "tyrosine 3-monooxygenase/tryptophan 5-monooxygenase activation protein, theta polypeptide" | 1.641011754 | 4.626315 |
| *ZC3H3* | zinc finger CCCH-type containing 3 | 1.735707677 | 4.626315 |
| *ZWILCH* | "Zwilch, kinetochore associated, homolog (Drosophila)" | 1.699396427 | 4.626315 |
