# Supplementary material for: Integrative Genomic Analyses Identify BRF2 as a Novel Lineage-Specific Oncogene in Lung Squamous Cell Carcinoma
Source: PLoS Med. 2010 Jul 27;7(7):e1000315. doi: 10.1371/journal.pmed.1000315 (PMC2910599; doi:10.1371/journal.pmed.1000315)
Supplement: Table S6 — BRF2 expression signature. (0.05 MB DOC) [file pmed.1000315.s012.doc]

**Table S5:** Raw qRT-PCR Data for NSCLC Cell Lines

| **Sample** | **Subtype** | **High-Level BRF2 Amplification** | **Average Threshold Cycle (Ct) BRF2 (Duplicates)** | **(Average Ct of 18S) - gene** |
| --- | --- | --- | --- | --- |
| H2122 | AC | No | 29.06752684 | 19.85829814 |
| H2347 | AC | No | 28.63241142 | 20.44297758 |
| HCC193 | AC | No | 28.24635077 | 19.93416649 |
| H1395 | AC | No | 27.40835672 | 21.21745197 |
| H2009 | AC | No | 28.8360165 | 21.73539205 |
| H1993 | AC | No | 28.55425511 | 20.38415562 |
| HCC4006 | AC | No | 31.09228349 | 22.98246889 |
| HCC2279 | AC | No | 29.61462185 | 21.49194505 |
| H2087 | AC | No | 28.30152644 | 19.34771139 |
| HCC78 | AC | No | 28.41332933 | 20.01162627 |
| HCC461 | AC | No | 27.92189898 | 19.59714154 |
| HCC1195 | AC | No | 27.39550363 | 20.03521307 |
| H1819 | AC | No | 27.322159 | 18.84645323 |
| H1648 | AC | No | 27.50537118 | 19.91216292 |
| HCC366 | AC | No | 27.81878734 | 20.27249214 |
| H3255 | AC | No | 28.20651325 | 20.65686577 |
| HCC2450 | SqCC | No | 28.23085247 | 19.75334058 |
| HCC15 | SqCC | No | 28.46420404 | 20.50868265 |
| HCC95 | SqCC | Yes | 27.14795231 | 17.87246371 |
| H520 | SqCC | Yes | 28.24238367 | 18.02467412 |
| Normal Lung | Normal | No | 30.55897836 | 20.91516717 |
