# Supplementary material for: Integrative Genomic Analyses Identify BRF2 as a Novel Lineage-Specific Oncogene in Lung Squamous Cell Carcinoma
Source: PLoS Med. 2010 Jul 27;7(7):e1000315. doi: 10.1371/journal.pmed.1000315 (PMC2910599; doi:10.1371/journal.pmed.1000315)
Supplement: Table S7 — Cellular functions enriched in NSCLC tumors with high BRF2 expression. (0.08 MB DOC) [file pmed.1000315.s013.doc]

**Table S7:** Cellular Functions Enriched in NSCLC Tumors with High *BRF2* Expression

| **Category** | **P-value*** | **Molecules** |
| --- | --- | --- |
| RNA Post-Transcriptional Modification | 0.0000017-0.0473 | FBL, CPSF6, RRP9, SNRPA, SFRS10, CSTF2T, LSM1, CPSF3, ACO1, EIF4EBP1 |
| Gene Expression | 0.000777-0.0339 | E2F6, GTF2F2, PTTG1, ACO1, EYA2, SNAPC5, EIF4EBP1 |
| Cell Cycle | 0.00247-0.0421 | E2F6, PAK2, EPOR, PTTG1, GSK3B, ASCL2 |
| Embryonic Development | 0.00247-0.0473 | PAK2, PRPF19, EPOR, PTTG1, PIK3CB, GSK3B, ASCL2, EYA2 |
| Amino Acid Metabolism | 0.00537-0.0421 | SLC7A1 |
| Behavior | 0.00537-0.0318 | GYS1, EPOR |
| Cancer | 0.00537-0.0473 | EPOR, PTTG1, TPI1, TUBB, BRF2, STK35, STAR, EIF4EBP1, E2F6, LSM1, CCT5, GSK3B, UNG, ASH2L, ALDOC |
| Carbohydrate Metabolism | 0.00537-0.0473 | HYAL2, GYS1, GSK3B, ACO1 |
| Cardiovascular Disease | 0.00537-0.0213 | GSK3B, OLR1 |
| Cardiovascular System Development and Function | 0.00537-0.0213 | PAK2, EPOR, GSK3B, OLR1 |
| Cell Death | 0.00537-0.0473 | YWHAQ (includes EG:10971), PRPF19, PAK2, EPOR, GSK3B, SH3KBP1, OLR1, BAG4, UNG |
| Cell Signaling | 0.00537-0.00537 | GSK3B |
| Cell-To-Cell Signaling and Interaction | 0.00537-0.0318 | CBLL1, ASCL2, OLR1 |
| Cellular Assembly and Organization | 0.00537-0.037 | PAG1, PTTG1, MID1, GFM1, EMD, POLS, GSK3B, TUBB, EIF4EBP1 |
| Cellular Compromise | 0.00537-0.0421 | GSK3B, TUBB, EIF4EBP1 |
| Cellular Development | 0.00537-0.0473 | EPOR, GSK3B, SHOX2, EYA2, OLR1, STAR |
| Cellular Growth and Proliferation | 0.00537-0.0473 | PRPF19, EPOR, EIF4EBP1 |
| Connective Tissue Development and Function | 0.00537-0.0473 | PAK2, PTTG1, GSK3B, SHOX2, UNG, EIF4EBP1 |
| DNA Replication, Recombination, and Repair | 0.00537-0.0473 | PRPF19, UNG, NUDT15 |
| Developmental Disorder | 0.00537-0.0265 | PTTG1, MID1 |
| Digestive System Development and Function | 0.00537-0.037 | EPOR, GSK3B, SHOX2 |
| Drug Metabolism | 0.00537-0.0213 | HYAL2, UNG |
| Endocrine System Disorders | 0.00537-0.0473 | PTTG1, STAR |
| Genetic Disorder | 0.00537-0.0473 | EPOR, MID1, TPI1, BTD, STAR |
| Hematological Disease | 0.00537-0.0399 | EPOR, TPI1, TUBB, EIF4EBP1 |
| Hematological System Development and Function | 0.00537-0.0473 | EPOR |
| Hepatic System Development and Function | 0.00537-0.0265 | EPOR, GSK3B |
| Metabolic Disease | 0.00537-0.00537 | TPI1, BTD |
| Nervous System Development and Function | 0.00537-0.0213 | EPOR, GSK3B, EIF4EBP1 |
| Nucleic Acid Metabolism | 0.00537-0.0107 | UNG, NUDT15 |
| Organ Development | 0.00537-0.0265 | EPOR, GSK3B, STAR |
| RNA Damage and Repair | 0.00537-0.00537 | FBL |
| Reproductive System Development and Function | 0.00537-0.0265 | EPOR, STAR |
| Reproductive System Disease | 0.00537-0.0473 | EPOR, PTTG1, LSM1, TPI1, GSK3B, BRF2, ASH2L, STAR |
| Respiratory Disease | 0.00537-0.037 | EPOR, PTTG1, GSK3B, TPI1, STK35, TUBB, ALDOC |
| Skeletal and Muscular System Development and Function | 0.00537-0.0421 | PAK2, EPOR, GSK3B |
| Small Molecule Biochemistry | 0.00537-0.0473 | HYAL2, SLC7A1, ALDH3B1, PIK3CB, ACO1, OLR1, NUDT15, UNG, STAR |
| Tissue Development | 0.00537-0.016 | EPOR, OLR1 |
| Tissue Morphology | 0.00537-0.0473 | EPOR, EIF4EBP1 |
| Tumor Morphology | 0.00537-0.016 | EIF4EBP1 |
| Vitamin and Mineral Metabolism | 0.00537-0.00537 | GSK3B |
| Immunological Disease | 0.00666-0.0399 | PTTG1, TPI1, TUBB |
| Cell Morphology | 0.0105-0.016 | PTTG1, EMD, OLR1 |
| Endocrine System Development and Function | 0.0107-0.0421 | STAR |
| Lipid Metabolism | 0.0107-0.0473 | PIK3CB, OLR1, STAR |
| Organ Morphology | 0.0107-0.0213 | EPOR, PTTG1, GSK3B |
| Organismal Injury and Abnormalities | 0.0107-0.016 | EPOR, OLR1, STAR |
| Cellular Movement | 0.016-0.0265 | GSK3B, OLR1 |
| Cellular Response to Therapeutics | 0.016-0.0213 | EPOR, GSK3B |
| Ophthalmic Disease | 0.016-0.0318 | EPOR |
| Skeletal and Muscular Disorders | 0.016-0.016 | E2F6, EIF4EBP1 |
| Gastrointestinal Disease | 0.0178-0.0466 | PTTG1, CCT5, GSK3B, TUBB, UNG |
| Protein Synthesis | 0.0203-0.0488 | EIF4H, DNPEP, EIF4EBP1 |
| Respiratory System Development and Function | 0.0213-0.0213 | EPOR |
| Dermatological Diseases and Conditions | 0.0234-0.0473 | YWHAQ (includes EG:10971), PAK2, GSK3B, BAG4 |
| Neurological Disease | 0.0265-0.0473 | EPOR, PTTG1, GSK3B, SH3KBP1, UNG |
| Inflammatory Disease | 0.0318-0.0318 | EPOR |
| Molecular Transport | 0.0318-0.0421 | SLC7A1, PIK3CB, OLR1 |
| RNA Trafficking | 0.0318-0.0318 | EIF4EBP1 |
| Hepatic System Disease | 0.0421-0.0421 | GSK3B |
| Organismal Development | 0.0421-0.0421 | EPOR, PIK3CB |

* The two significance values refer to the range of specific sub-functions encompassed by the main function listed
