# Supplementary material for: Integrative Genomic Analyses Identify BRF2 as a Novel Lineage-Specific Oncogene in Lung Squamous Cell Carcinoma
Source: PLoS Med. 2010 Jul 27;7(7):e1000315. doi: 10.1371/journal.pmed.1000315 (PMC2910599; doi:10.1371/journal.pmed.1000315)
Supplement: Table S8 — High-level amplifications associated with SqCC tumors with and without BRF2 activation. (0.04 MB DOC) [file pmed.1000315.s014.doc]

**Table S8:** High-level Amplifications Associated with SqCC Tumors with and without BRF2 Activation^1^

| **Cytoband** | ***q* value** | **Wide peak boundaries^2^** | **Candidate target** |
| --- | --- | --- | --- |
| **SqCC Tumors with *BRF2* Copy Number Increase** | | | |
| 3q26.2 | 0.03 | 168.23-194.13 | *SOX2* |
| 8p12 | 2.90E-06 | 35.46-40.07 | *BRF2* |
| 11p13 | 0.12 | 31.01-37.54 | *CD44* |
| 12q14.1 | 0.16 | 59.41-64.42 | *CDK4^3^* |
| 12q15 | 0.17 | 62.99-67.60 | *MDM2* |
| **SqCC Tumors without *BRF2* Copy Number Increase** | | | |
| 1p34.2 | 0.06 | 38.93-40.79 | *MYCL1* |
| 3q26.33 | 2.84E-09 | 182.10-184.73 | *SOX2* |
| 5p14.1 | 0.16 | 8.22-43.13 | *TERT^3^* |
| 7p11.2 | 0.0003 | 54.16-56.13 | *EGFR* |
| 8q24.21 | 0.12 | 120.28-132.17 | *MYC* |
| 9p13.3 | 0.08 | 32.56-36.18 | N/A |
| 14q32.2 | 0.20 | 96.53-100.63 | N/A |
| 20p12.1 | 0.20 | 10.36-20.34 | N/A |

^1^ 92 SqCC tumors were separated based on their *BRF2* copy number status (38 with *BRF2* copy number increase and 54 without) and *aCGH-Smooth* was used to smooth ratio values and identify copy number breakpoints for each sample as described in the Methods section. The resulting segments and ratio values were then analyzed using the *Genomic Identiﬁcation of Signiﬁcant Targets in Cancer* (*GISTIC*) method [Beroukhim *et al*. *Proc Natl Acad Sci U S A* 104:20007-12, 2007] to determine regions of significant amplification in each group. Analysis was performed using *Gene Pattern* software ([www.broad.mit.edu/cancer/software/genepattern/](http://www.broad.mit.edu/cancer/software/genepattern/)) with default settings with the exception of the amplification threshold = 0.6. In addition, known regions of copy number variation in the normal population [Wong *et al. Am J Hum Genet* 80:91-104, 2007] were removed during the analysis. Lastly, only focal regions of amplification (wide peak region < chromosome arm in size) are reported.

Tumors without *BRF2* increase harboured amplifications in other well-known lung cancer oncogenes including *EGFR* and *MYC*. In contrast, SqCCs with *BRF2* amplification also displayed amplification of *MDM2*, further highlighting the potential importance of p53 inactivation in BRF2-mediated tumorigenesis. Of interest, both SqCC subsets contain amplification of chromosome arm 3q, which has previously been shown to be a frequent event in SqCC tumorigenesis. Recently, *SOX2* has been identified as the target of this amplification and lineage-survival oncogene in lung and esophageal SqCCs [Bass *et al. Nat Genet* 41:1238-42, 2009]. Together, this preliminary investigation suggests that SqCC lung tumors with amplification of *BRF2* may represent a distinct subset of SqCC that develop through a unique genetic pathway, independent of common lung cancer oncogenes such as *EGFR* and *MYC*.

^2^Boundaries are reported as the Mbp position on the respective chromosome.

^3^Gene immediately adjacent to wide-peak region.
